# Transcriptional cofactors Ski and SnoN are major regulators of the TGF-β/Smad signaling pathway in health and disease

**DOI:** 10.1038/s41392-018-0015-8

**Published:** 2018-06-08

**Authors:** Angeles C. Tecalco-Cruz, Diana G. Ríos-López, Genaro Vázquez-Victorio, Reyna E. Rosales-Alvarez, Marina Macías-Silva

**Affiliations:** 1 Instituto de Investigaciones Biomédicas at Universidad Nacional Autónoma de México, Mexico city, 04510 Mexico; 2 Instituto de Fisiología Celular at Universidad Nacional Autónoma de México, Mexico city, 04510 Mexico; 3 Facultad de Ciencias at Universidad Nacional Autónoma de México, Mexico city, 04510 Mexico

## Abstract

The transforming growth factor-β (TGF-β) family plays major pleiotropic roles by regulating many physiological processes in development and tissue homeostasis. The TGF-β signaling pathway outcome relies on the control of the spatial and temporal expression of >500 genes, which depend on the functions of the Smad protein along with those of diverse modulators of this signaling pathway, such as transcriptional factors and cofactors. Ski (Sloan-Kettering Institute) and SnoN (Ski novel) are Smad-interacting proteins that negatively regulate the TGF-β signaling pathway by disrupting the formation of R-Smad/Smad4 complexes, as well as by inhibiting Smad association with the p300/CBP coactivators. The Ski and SnoN transcriptional cofactors recruit diverse corepressors and histone deacetylases to repress gene transcription. The TGF-β/Smad pathway and coregulators Ski and SnoN clearly regulate each other through several positive and negative feedback mechanisms. Thus, these cross-regulatory processes finely modify the TGF-β signaling outcome as they control the magnitude and duration of the TGF-β signals. As a result, any alteration in these regulatory mechanisms may lead to disease development. Therefore, the design of targeted therapies to exert tight control of the levels of negative modulators of the TGF-β pathway, such as Ski and SnoN, is critical to restore cell homeostasis under the specific pathological conditions in which these cofactors are deregulated, such as fibrosis and cancer.

## Growth suppressors: Antagonistic proteins offer drug targets for fibrosis and cancer

Proteins that repress molecular signaling through the transforming growth factor-beta (TGF-β) pathway offer promising targets for treating cancer and fibrosis. Marina Macías-Silva and colleagues from the National Autonomous University of Mexico in Mexico City review the ways in which a pair of proteins, called Ski and SnoN, interact with downstream mediators of TGF-β to inhibit the effects of this master growth factor. Aberrant levels of Ski and SnoN have been linked to diverse range of diseases involving cell proliferation run amok, and therapies that regulate the expression of these proteins could help normalize TGF-β signaling to healthier physiological levels. For decades, drug companies have tried to target the TGF-β pathway, with limited success. Altering the activity of these repressors instead could provide a roundabout way of remedying pathogenic TGF-β activity in fibrosis and oncology.

## Introduction

**Fig. 1:**
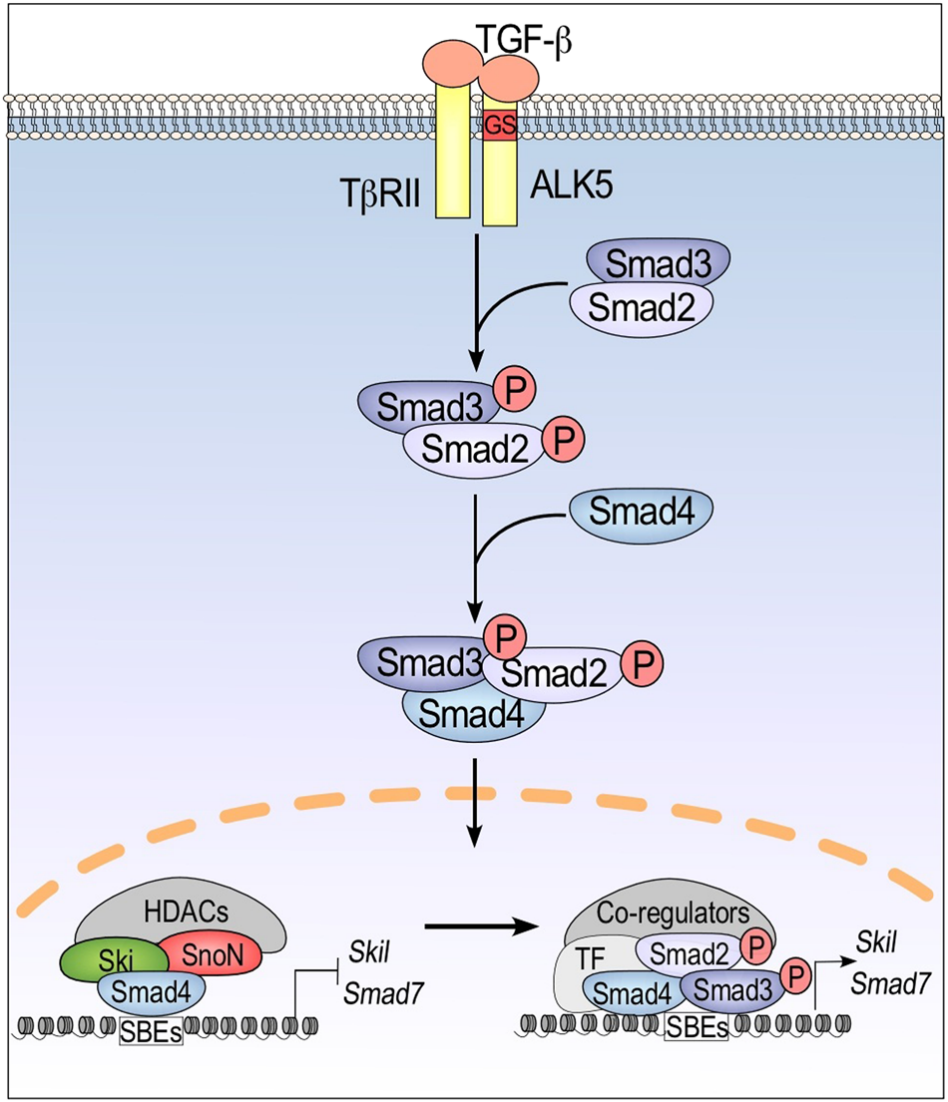
Regulation of the TGF-β/Smad signaling pathway by Ski and SnoN. In the absence of TGF-β, the Ski and SnoN proteins interact with Smad4 to inhibit the expression of TGF-β target genes, such as *Smad7* and *Skil*, by recruiting other corepressors and HDACs to their promoters. Then, TGF-β induces the phosphorylation of Smad2/3 proteins to form the R-Smad/Smad4 complex, which associates with specific transcription factors and cofactors to modulate the expression of its target genes

The transforming growth factor-beta (TGF-β) superfamily comprises a large group of related growth factors, such as TGF-βs, activins, inhibins, bone morphogenetic proteins (BMPs), myostatin, nodal, lefty, anti-Müllerian hormone/Müllerian inhibiting substance (AMH/MIS), and growth and differentiation factors (GDFs).^1^ This family of cytokines and differentiation factors has major pleiotropic activities in development and tissue homeostasis and exerts altered functions in diverse pathologies.^2–5^ Upon ligand binding to type II and type I receptors, the active ligand-heterotetrameric receptor complex signals through downstream transcriptional factors named Smads (Fig. 1).^6–10^ The Smad protein family is divided into three groups: R-Smad (Smad1, Smad2, Smad3, Smad5, and Smad8), co-Smad (Smad4), and I-Smad (Smad6 and Smad7). Receptor-regulated Smads (R-Smad) have an SSXS motif in their C-terminal region that is phosphorylated by type I receptors; R-Smad phosphorylation (p-R-Smad) allows their association with Smad4.^11–18^ After translocation of the p-R-Smad/Smad4 heterotrimeric complex into the nucleus, Smads associate with other transcriptional factors and coregulators to regulate the expression of specific target genes. TGF-β signaling regulates the transcription of >500 genes, which may contain one or more Smad binding elements (SBEs) in their promoter region (Fig. 1). Multiple Smad-interacting transcription factors may cooperate with Smads to modulate-specific target gene expression, depending on the cellular type, in both physiological and pathological conditions.^19,20^

The luminescence-based mammalian interactome mapping (LUMIER) dataset comprises >100 proteins associated with the TGF-β receptor complex or with Smad proteins; although, the biological relevance of each interaction remains to be elucidated. The LUMIER dataset is available to support the investigation of TGF-β signaling networks in different cell types.^21^ Furthermore, the activity and stability of Smad proteins are regulated by diverse post-translational modifications (PTMs), such as phosphorylation, acetylation, ubiquitination, sumoylation, and parylation; thus, the variety of Smad isoforms generated by PTMs increases the complexity of TGF-β signal transduction networks.^20,22–24^ The TGF-β signaling pathway is also modulated by multiple mechanisms, including the actions of diverse positive and negative regulators, which orchestrate temporal and spatial actions of TGF-β signaling.^25,26^ TGF-β signals may also regulate the expression of their own regulators, generating negative feedback loops to control the magnitude and duration of TGF-β signaling. Among the negative regulators of TGF-β signals are TGIF, Ski, and SnoN transcriptional cofactors, which can inhibit Smad transcriptional activity to exert differential regulation of TGF-β-targeted gene expression (Fig. 1).^27–30^

## Ski and snon are major negative regulators of the Tgf-β signaling pathway

The homologous Ski and SnoN proteins belong to the Ski protein family; they are mainly localized in the nucleus and may induce cell transformation when overexpressed.^29–33^ The Ski protein family includes several members, such as Ski, SnoN, SnoN2, SnoI, SnoA, CORL-1, DACH1/Dach1, DACH2/Dach2, Fussel-15 (SKOR1; LBXCOR1), Fussel-18 (SKOR2; CORL-2), DAF-5, Dachshund, and Fusel.^34–48^ Presently, Ski and SnoN have been identified as key transcriptional corepressors of Smad proteins and exhibit vital biological functions by controlling the TGF-β/Smad signaling pathway during embryogenesis and tissue homeostasis.^29,30,33^ Ski and SnoN act as Smad corepressors by indirect binding to the consensus sequence 5′-GTCTAGAC-3′ (known as SBE) through their association with Smad proteins.^49,50^ According to the current model, Ski and SnoN block TGF-β signaling by forming an inhibitory complex with Smad proteins on SBEs of the TGF-β-target gene promoters, and such complexes recruit histone deacetylases (HDACs) and additional corepressors to inhibit gene expression (Fig. 1).^29,30,33^

Ski functions rather as a transcriptional coregulator since it binds only to DNA in a complex with other transcriptional factors, which is essential for target-gene activation or repression.^51–55^ Notably, Ski acts as a potent inhibitor of transcriptional factors, such as Smads and Gli3, in mouse embryos through its association with HDACs, NCoR, and mSin3A.^51,56–58^ The Ski protein disturbs TGF-β signaling by competing with the CREB-binding protein (CBP) for the binding of activated Smads.^59^ Ski may also repress the expression of some BMP target genes by its interaction with the homeodomain-interacting protein kinase 2 (HIPK2) in mouse myoblasts.^60^ Additional examples include the inhibition of Smad2 transcriptional activity via the interaction with Ski and c-Jun;^61^ inactivation of the vitamin-D-receptor (VDR) signaling pathway by the Ski/NCoR complex;^62^ inhibition of the retinoic acid (RA) signaling and the transcription factor PU.1 expression by Ski/HDAC3;^63,64^ decreased *Ctip2* gene expression by the Ski/HDAC1 and Satb2/MTA2 complexes;^65,66^ Ski inhibition of GATA1 binding to DNA and its transcriptional activity;^67^ Ski and SIRT1 inhibition of p53 actions;^68^ and Hippo pathway inhibition through the recruitment of NCoR by Ski to the TEAD/TAZ protein complex.^69^ Transcriptional gene silencing is also mediated by the interactions of Ski with other factors, such as PRMT5, HDAC3, Rb, MeCP2, and Mad, as well as with the thyroid hormone receptor β (TRβ).^54,70–72^

Likewise, the SnoN protein also functions as a transcriptional coregulator; it associates with Smads to inhibit gene expression, including the silencing of its own gene *Skil* (Fig. 1).^73^ To date, a few genes have been identified as targets of the SnoN/Smad complex, including those encoding Smad7, SnoN, FGF8, GSC, MIXL1, and AFP.^54,73–76^ Other genes that are regulated by SnoN encode miR720, miR274A, miR1274B, ADAM12, PLSCR1, Ccd1, and pS2.^77–83^

Ski function as a coactivator became evident after the demonstration of its association with the nuclear factor 1 (NF1) family of transcription factors.^84,85^ Ski favors β-catenin signaling to promote the expression of *Mitf* and *Nr-CAM* genes in melanoma.^86^ Ski also favors *myogenin* gene expression and myogenesis by forming a complex with specific transcription factors, such as MyoD,^87^ as well as with Six1 and Eya3.^88^ SnoN can also act as a coactivator for Smads and for other transcriptional factors, but this ability depends on specific target genes;^77,78^ for instance, SnoN is a coactivator for estrogen receptors (ER) and enhances the estrogen-signaling pathway in breast cancer cells.^80^ Nevertheless, more studies are required to reveal the versatility of Ski and SnoN mechanisms to control gene transcription.

### Structure and regulation of *Ski* and *Skil* genes

**Fig. 2:**
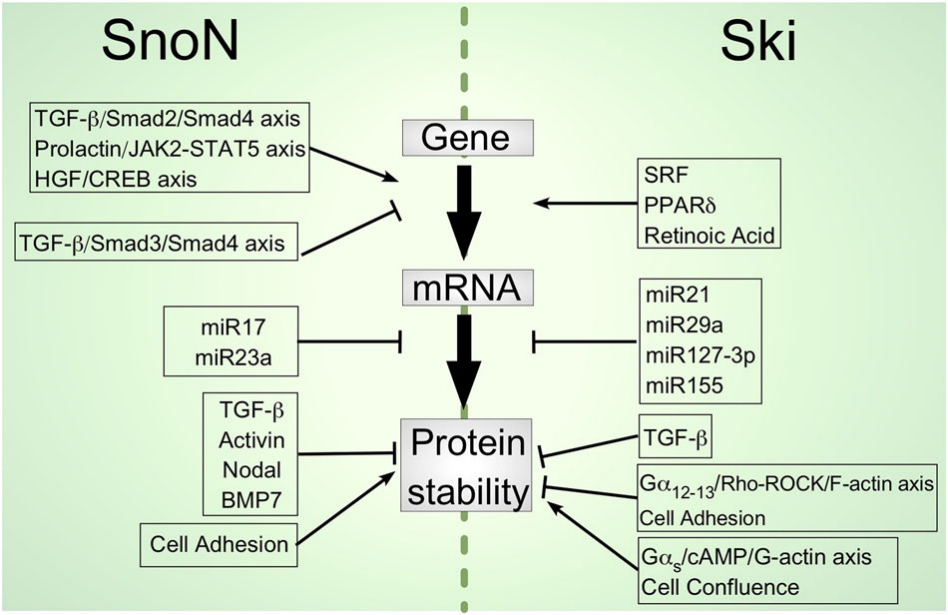
Regulation of Ski and SnoN expression by different mechanisms. Several signaling pathways and factors induce *Ski* and *Skil* gene expression; whereas, the TGF-β signaling pathway enhances or inhibits *Skil* gene expression via Smad2/4 or Smad3/4, respectively. Moreover, some miRNAs control the mRNA levels of these genes. The Ski and SnoN protein levels are also regulated by different signals

The viral form *v-Ski* was identified as a gene inserted into the avian Sloan-Kettering retroviruses (SKVs) genome.^89,90^ Later, a homologous proto-oncogene was identified in chicken and denoted *c-Ski* (cellular gene).^90^ Ski homologs have been detected in several vertebrate species from fish to humans.^90–94^ The human *Ski* gene is localized at chromosome 1 and generates at least two transcripts that encode a 728-amino acid (aa) protein.^91,95^ Thus, there is no evidence of Ski mRNA splicing, and it appears that Ski transcript differences rely on the length of the 3′-UTR (untranslated region).^91,96^ Regulation of *Ski* gene expression has been poorly studied; however, some factors or signals have been identified as potential regulators of Ski expression, such as serum-response factor (SRF), peroxisome proliferator-activated receptor δ (PPARδ), and RA signaling (Fig. 2).^97–100^ Recently, it has been reported that the *Ski* gene is hypermethylated and silenced in human primary lung cancer tissues, supporting Ski function as a tumor suppressor.^101^ Ski mRNA levels are also modulated by different miRNAs, such as miR-21, miR-29a, miR-155, and miR-127-3p (Fig. 2).^102–107^

**Fig. 3:**
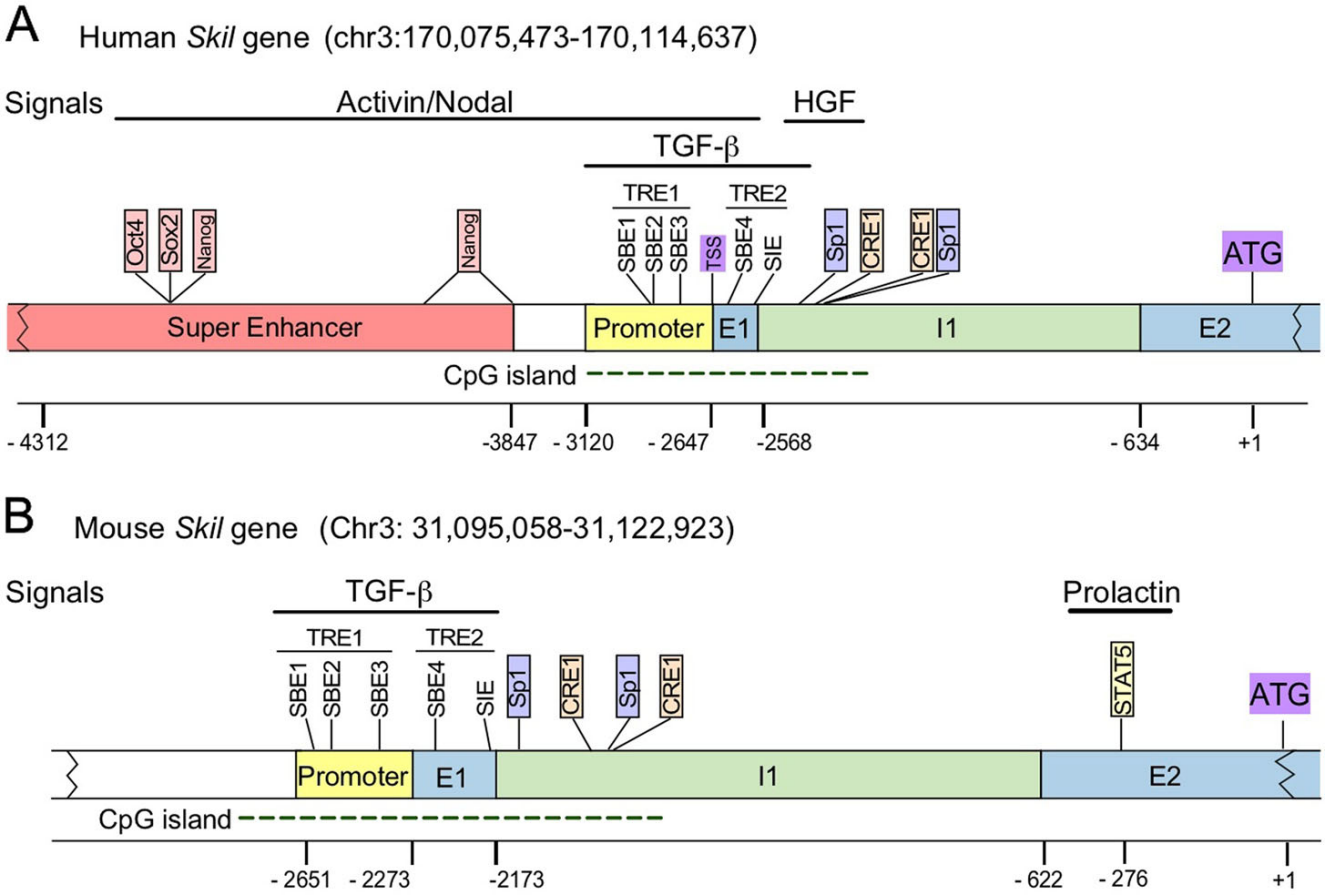
Regulatory regions of human and mouse *Skil* genes. The *Skil* gene promoter includes two TGF-β-responsive elements (TRE) containing several Smad binding elements (SBEs and SIE). The human *Skil* gene superenhancer region contains binding sites for Oct4, Sox2, and Nanog proteins. The mouse *Skil* gene promoter includes the SBEs and SIE, as well as a binding site for STAT5 that is controlled by prolactin. The positions of response elements and transcription start site (TSS) are indicated with respect to the ATG (+1)

Human and mouse *Skil* genes are localized on chromosome 3 and encode for all SnoN isoforms. There is a limited characterization of the 5′ end regulatory sequences of both human and mouse *Skil* genes, including promoters and enhancers, due to the complex organization and modulation of these genes (Fig. 3). Both human and mouse *Skil* genes harbor two TGF-β-responsive elements (TRE) localized ~2 kb upstream of the translation starting site (ATG). TRE1 is localized in the core promoter and formed by three SBE sequences: SBE1, SBE2, and SBE3,^73,108^ whereas TRE2 is localized at exon 1 and contains the SBE4 and a Smad inhibitory element (SIE); all the SBEs are recognized by the Smad2/Smad4 complex to induce *Skil* gene expression, whereas SIE is recognized by the Smad3/Smad4 complex, which inhibits *Skil* gene expression (Fig. 3).^73,108^ Regarding the 5′-UTR of *Skil* mRNA, E1 (exon 1 with 170 bp) and a fragment of E2 (exon 2 with 633 bp) encode for the 5′-UTR of *Skil* mRNA and are separated by a large intron (1.9 kb).^73^

The TGF-β signaling pathway controls the expression of the *Skil* gene through diverse mechanisms: under basal conditions, the SnoN/Ski/Smad4 complex binds and represses the *Skil* gene promoter; then, after a short period of TGF-β stimulation, SnoN and Ski protein degradation occurs via the proteasome; whereas, the activated Smad2/Smad4 complex replaces the SnoN/Ski complex on the *Skil* gene promoter and promotes its induction. Both SnoN mRNA and protein levels are increased after TGF-β treatment for ~1.5 h. Subsequently, a negative feedback loop is generated, in which the SnoN protein binds the *Skil* gene promoter to inhibit its own expression.^73^ Interestingly, human *Skil* gene also contains a super-enhancer localized ~3.6 kb upstream from the ATG (+1) and ~1 kb upstream from the TRE1; the coordinated regulation of *Skil* super-enhancer by Oct4/Sox2/Nanog (OSN) transcriptional factors and *Skil* promoter by activated Smads seems to be essential for the maintenance of stem cell pluripotency (Figs. 2 and 3).^74^

TGF-β/Smad, activin, nodal, BMP7, HGF/CREB, and prolactin/STAT5 are the best-known signals that regulate *Skil* gene expression at the transcriptional level (Fig. 2).^73,109–113^ Other less studied pathways also appear to regulate *Skil* gene expression, such stimulation of the PI3K/Akt pathway with arsenic trioxide (As_2_O_3_), and inhibition of Smad3 phosphorylation with endocrine disrupting chemicals (EDC), such as 4-nonylphenol (NP) and bisphenol A (BPA).^114,115^ Furthermore, *Skil* expression is also regulated by miRNAs: miR-17 and miR-23a are potential regulators of the 3′-UTR of human *Skil* mRNA (Fig. 2);^116,117^ interestingly, miR-17 is a member of the miR-17-19-130 superfamily targeting tumor suppressors, such as the TGF-β, PI3K, and p53 pathways.

### Molecular structure and PTMs of the Ski and SnoN proteins

**Fig. 4:**
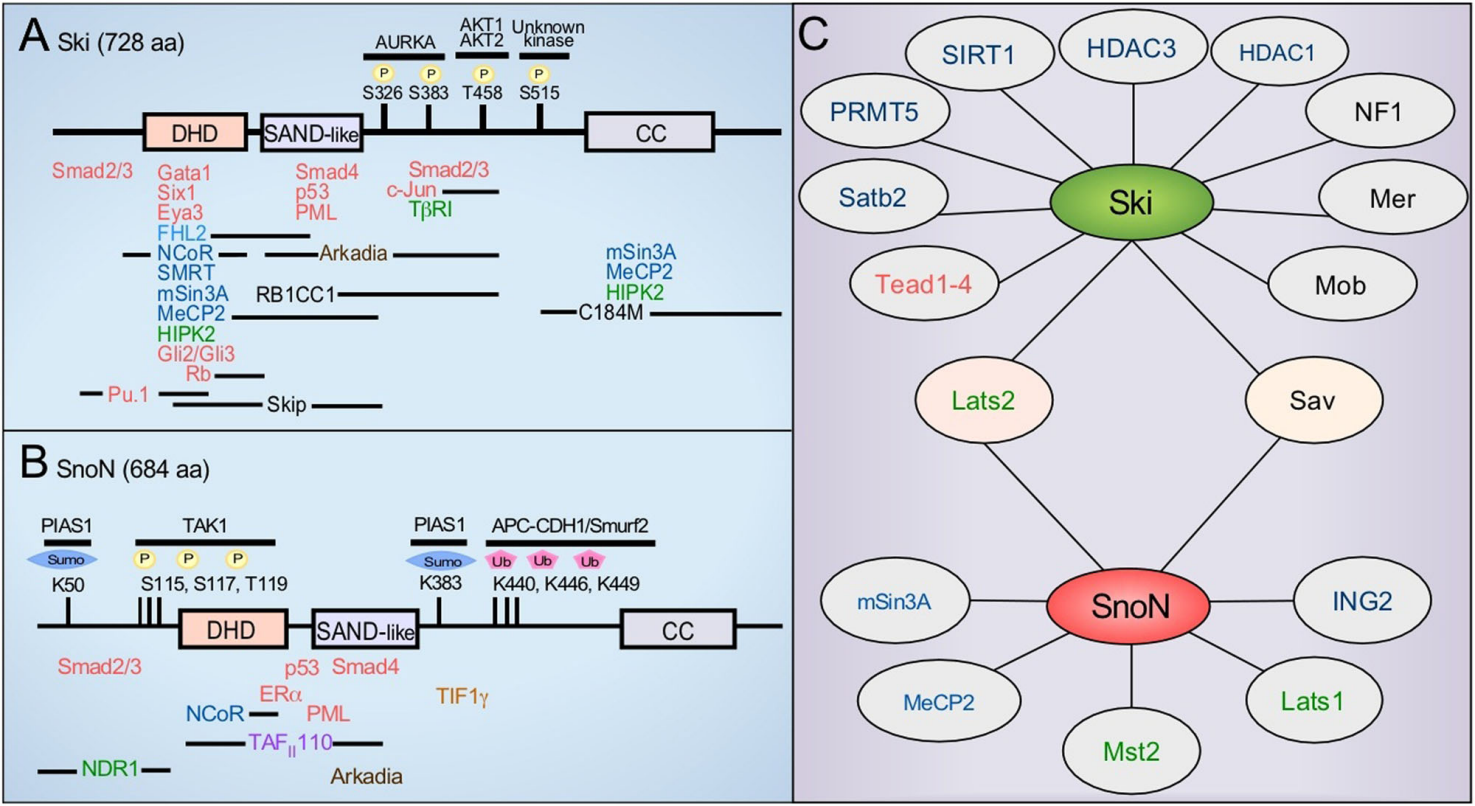
Molecular structure, posttranslational modifications, and protein–protein interactions of the Ski and SnoN proteins. **a**, **b** The domains that define the Ski and SnoN proteins include: Dachshund homology domain (DHD), SAND-like domain, and coiled-coil domain (CC). Both the Ski and SnoN proteins are regulated by PTMs, as catalyzed by several enzymes at specific residues that are indicated. Known regions of interaction with some partners for Ski and SnoN are indicated. **c** Several proteins are also partners for Ski and/or SnoN but the interacting domains are not identified. Kinases are shown in green, transcriptional coregulators in blue, transcription factors in red, and other proteins in black

The human Ski protein has 728 aa and three main domains (Fig. 4). The N-terminal Ski-dachshund homology domain (Ski-DHD) comprises a region of residues 91–192 with a folding pattern of mixed α/β structures; this domain has lost the ability to bind directly to DNA, but instead behaves as an interacting domain that binds R-Smads and other transcriptional regulators, such as NCoR and Skip.^51,56,58,71,118–121^ The Ski protein also has a SAND-like domain (residues 219–312) that includes an extended I-loop motif that is responsible for Smad4 binding (Fig. 4).^122^ Two other N-terminal regions in the Ski protein include a proline-enriched stretch (between residues 61 and 89) and the transformation domain that is localized in the first 304 residues; this last domain is responsible for some Ski activities, such as cell transformation and transcriptional gene repression.^49,50,123,124^ The Ski- and SnoN-DHD crystal structure reveals that the transformation domain is important for recruiting multiple partners (Fig. 4).^121,125,126^ The Ski protein differs at its C-terminus sequence, where it has a helical domain that forms a coiled-coil (CC) region constituting two motifs: a region of five tandem repeat (TR) motifs with 25 residues in each, and a leucine zipper (LZ) motif of six heptad repeats; both structural motifs are implicated in the formation of Ski homodimers and Ski/SnoN heterodimers.^124,127,128^

SnoN is a protein of 684 aa with four conserved domains in its N-terminus: minimal transformation domain, SAND-like domain, DHD domain, and Smad-binding domain. The C-terminus of the SnoN protein is variable and contains a domain for SnoN homodimerization and Ski/SnoN heterodimerization (Fig. 4).^122,127,129–131^ Four SnoN isoforms have been identified: SnoN (non-alu-containing), SnoN2, SnoA (alu-containing), and SnoI (insertion). All SnoN isoforms result from alternative splicing and almost all are ubiquitously expressed, except SnoI, which is mainly expressed in skeletal muscle. SnoN2 differs from SnoN by a deletion of 138 nucleotides; whereas, SnoI and SnoA encode truncated proteins of 399 and 415 aa, respectively. Although both SnoI and SnoA proteins lack the dimerization domain, only SnoA has a unique domain in its C-terminal region.^91,132,133^ The four SnoN isoforms have been identified in *Drosophila* and humans; whereas, SnoN and SnoN2 are the only isoforms expressed in mice.^133–135^ Nevertheless, the localization, regulation, and function of all SnoN isoforms have not yet been completely revealed.

Ski was initially described as a phosphorylated protein in serine residues.^32^ Growth factors and hormones, such as hepatocyte growth factor (HGF), insulin-like growth factor-1 (IGF-1), and insulin promote Ski protein degradation by inducing its phosphorylation at the Thr458 residue by AKT kinase.^136^ The Ski protein residue Ser515 is also phosphorylated, but the kinase involved has not been identified; however, mutation of Ser515 does not affect Ski activity as a transcriptional corepressor.^137^ Ski also interacts with Aurora A kinase (Aurka) through its C-terminal region; Aurka phosphorylates Ski at Ser326 and Ser383 to decrease Ski stability (Fig. 4).^138,139^ By contrast, TAK1 kinase promotes SnoN protein phosphorylation at residues Ser115, Ser117, and Thr119 after TGF-β treatment, which causes polyubiquitination and posterior degradation of the SnoN protein (Fig. 4).^140^

**Fig. 5:**
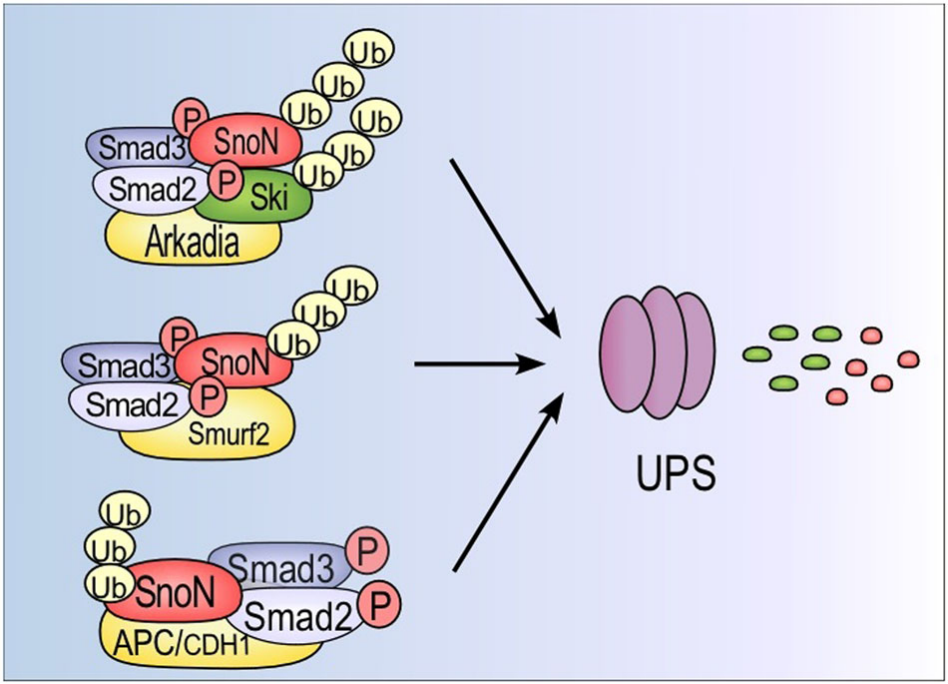
Regulation of Ski and SnoN protein stability by the TGF-β/Smad signaling pathway. TGF-β induces Ski and SnoN protein degradation by the ubiquitin-proteasome system (UPS) after 15–30 min of treatment because activated R-Smads are adapters for ubiquitin E3-ligases APC, Smurf2, and Arkadia

Ski and SnoN protein polyubiquitination causes their degradation via the ubiquitin-proteasome system (UPS). To date, three main E3 ubiquitin-ligases interacting with SnoN have been identified: the anaphase-promoting complex (APC), Smad-ubiquitination-related-factor 2 (Smurf2), and Arkadia all catalyze SnoN polyubiquitination (Figs. 4 and 5).^141–148^ The Smurf2 WW domains interact with the Smad2 PY motif, and this Smurf2/Smad2 complex recruits the SnoN protein.^141,149^ SnoN polyubiquitination by Smurf2 requires two segments of the SnoN protein: one region (residues 1–97) binds the Smad2 MH2 domain, and the second region (residues 366–684) harbors the target lysines for ubiquitination.^141^ APC binds Smad2 and Smad3 proteins to promote SnoN polyubiquitination; in this case, SnoN has a D-box motif between residues 164 and 172 (RLCLPQVLN), which is equivalent to the destruction box of most APC substrates. SnoN protein residues K440, K446, and K449 are the targets for polyubiquitination by APC (Figs. 4 and 5),^141–143,150^ whereas in the case of Ski, the polyubiquitinated lysine residues have not yet been identified. Arkadia was identified as a major positive regulator of TGF-β signaling because it causes the downregulation of Smad7, Ski, and SnoN negative regulators. Arkadia interacts with Ski (at residues 211–490) and SnoN (at residues 263–355) and catalyzes their polyubiquitination in a manner dependent on the recruitment of activated Smad2/Smad3.^144–146,148^ Recently, it was shown that Smurf2 induces Smad4 monoubiquitination to inhibit its function as mediator of TGF-β signaling, whereas formation of the Ski/Smad4 complex blocks Smad4 monoubiquitination.^151^

SnoN protein sumoylation requires the participation of Ubc9 (SUMO E2-conjugating enzyme) and PIAS1 or TIF1γ (SUMO E3 ligases); the addition of SUMO occurs at the K50 and K383 residues of the SnoN protein (Fig. 4).^152–155^ This SnoN sumoylation is important for SnoN-mediated repression of key genes, such as *myogenin*, in the C2C12 myoblast cell line and for the negative regulation of myogenesis.^152,153^ Sumoylation of SnoN does not appear to affect its function as a transcriptional corepressor of Smad since the sumoylated SnoN protein can still inhibit the TGF-β-dependent repression of E-cadherin during the epithelial-mesenchymal transition (EMT) process. TGF-β signaling inhibits SnoN sumoylation by decreasing PIAS1 levels during EMT of the mammary epithelial NMuMG cell line.^154^ By contrast, sumoylation of SnoN1 by TIF1γ is necessary during acinar morphogenesis of NMuMG cells to inhibit EMT induced by TGF-β.^155^ TIF1γ catalyzes only the sumoylation of SnoN1, but not of SnoN2. Regarding Ski, there is lack of evidence about its sumoylation, although Ski interacts with the Ubc9 enzyme to enhance its activity. Thus, Ubc9 promotes MDM2 mono-sumoylation, which in turn promotes p53 protein polyubiquitination and degradation.^156^

## Regulation of subcellular localization and stability of the Ski and Snon proteins

The Ski and SnoN proteins can be localized in both the cytoplasm and nucleus; intriguingly, Ski and SnoN appear to have an exclusive nuclear localization in most cancer cell lines. Ski and SnoN have non-homologous nuclear localization signals (NLS); the SnoN NLS is localized at its N-terminus and depends on residues K30 and K31,^157^ whereas residues 452–458 (sequence PRKRKLT) comprise the Ski NLS.^158^ Ski and SnoN mutants in their respective NLSs exhibit high stability and may sequester Smad proteins in the cytoplasm.^157,158^

Some proteins retain Ski and SnoN in the cytoplasm; for instance, the interaction of Ski with the C184M protein in the cytoplasm may block Smad2 translocation to the nucleus despite the activation of TGF-β signaling, in both the liver and lenses.^159,160^ The Ski and SnoN proteins can also interact with the TβRI receptor (or ALK5) in the cytoplasm, inducing failure of the R-Smad/Smad4 complex to translocate to the nucleus.^161^ The Ski protein was found first in both nuclear and cytoplasmic fractions of melanoma cells.^162,163^ SnoN levels are modified during mouse mammary gland development; however, the localization of SnoN remains cytoplasmic in normal human breast cells, but not in breast cancer tissues.^157,164,165^ Vitamin C treatment decreases cytosolic Ski protein levels in rat kidney mesangial cells.^166^

In the peripheral nervous system, nuclear Ski regulates Schwann cell proliferation and differentiation by promoting normal myelination of axons.^167^ The TGF-β signaling may promote Ski relocalization into the cytoplasm, but without any protein degradation in Schwann cells; thus, the Ski protein is localized at early endosomes to sequester the phosphorylated retinoblastoma protein (pRb).^168^ TGF-β signaling also promotes Ski protein shuttling from the cytoplasm to the nucleus in cardiac myofibroblasts,^169^ whereas MG132 treatment causes accumulation of the Ski protein in the cytoplasm of human cervical cancer HeLa cells.^158^ Furthermore, the Ski and SnoN proteins associate with members of the Hippo pathway in the human mammary epithelial cells MDA-MB-231 and MCF10A; if these cells are grown at a high density, then Lats2 kinase promotes a decrease in the SnoN protein levels.^69,170^

In the liver, the Ski protein has been localized in multivesicular endosomes (MVE); recently, we reported that cytoplasmic Ski is present in lipid raft-rich endosomes of normal hepatocytes and co-localizes with markers of multivesicular bodies, such as CD63 and Alix. Intriguingly, TGF-β signaling and proteasome inhibition by MG132 promote the localization of the Ski protein at these lipid raft-rich vesicles.^171^ The Ski and SnoN proteins are localized in both the cytoplasm and nucleus of C9 cells (rat hepatocyte clone 9) and primary rat hepatocytes.^172^ In this case, the stability of the Ski and SnoN proteins is regulated by actin-cytoskeleton dynamics (Fig. 2).^171,172^ The Ski protein levels are differentially modulated by GPCR (G-protein-coupled receptors) signals, i.e., GPCR signals associated with the G_12/13_/Rho/ROCK axis, which promote actin polymerization, downregulating the Ski protein, whereas GPCR signals associated with the G_s_/adenylate cyclase/cAMP axis, leading to the depolymerization of actin filaments, stabilizing the Ski protein. By contrast, the SnoN protein is stabilized by signals that promote actin polymerization in hepatocytes.^171–173^ Interestingly, Ski and SnoN regulation mediated by actin polymerization dynamics is lost in hepatoma cells, affecting notably the TGF-β signaling outcome.^172^ Other studies have shown that cAMP also increases the Ski protein levels, but in rat Schwann cells.^168^ Therefore, it is clear that cell density, polarity, and actin-cytoskeleton dynamics regulate Ski and SnoN protein stability, as well as their subcellular localization (Fig. 2).

The Ski and SnoN proteins stability can change during the cell cycle since protein levels decay rapidly during the transition from the G1 to S phases of the cell cycle, and both proteins are accumulated in the G2 phase and mitosis. The Ski protein is polyubiquitinated and degraded during the G1 phase, whereas it is temporally stabilized by phosphorylation during mitosis.^143,174,175^ The Ski protein may bind α- and γ-tubulin, and this interaction mediates Ski localization at the mitotic spindle and centrosomes during the cell cycle.^175^ Ski localization in the mitotic spindle is important in chromosome segregation; loss of both *Ski* alleles (Ski −/−) causes aneuploidy in mouse embryonic fibroblasts (MEFs).^176^ The Ski C-terminal domain is important for its co-localization with Aurka in centrosomes; this association regulates centrosomal amplification and multipolar mitosis in MEFs.^138,139^ Intriguingly, the number of complexes between Ski and SnoN with R-Smads appears to increase during mitosis.^177^ The role of Ski and SnoN during cell proliferation is unclear, thus meriting further studies.

## Role of Ski and Snon in development and tissue homeostasis

**Fig. 6:**
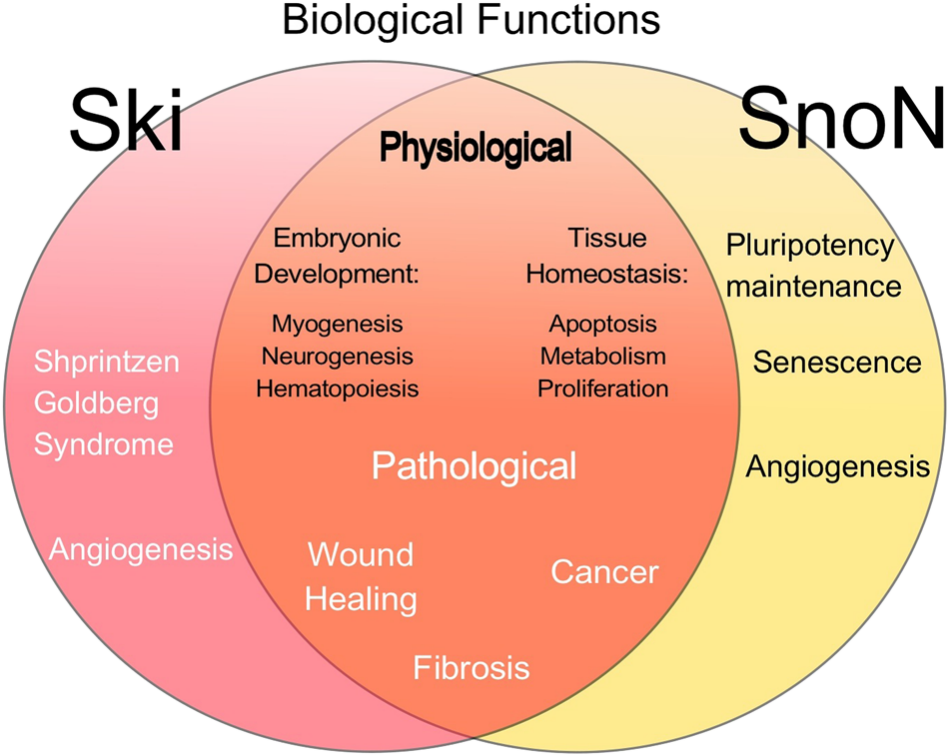
Ski and SnoN functions in health and disease. The specific or common biological functions of Ski and SnoN are linked to some physiological (black text) or pathological processes (white text)

TGF-β family members play crucial roles in regulating embryo development and tissue homeostasis. During these physiological processes, Ski and SnoN are strongly involved in controlling the spatio-temporal effects of the TGF-β pathway, as well as the magnitude and duration of the TGF-β signal. Ski and SnoN may also simultaneously regulate other signaling pathways during embryonic development or tissue homeostasis (Fig. 6). The role of SnoN during development has been partially determined based on the varied phenotypes exhibited by *Skil* knockout (KO) mice.^178,179^ Two *Skil* KO mice were generated by the deletion of exon 1: one of them resulted in embryo lethality;^178^ whereas, the other was viable but showed alterations in T-cell functions.^179^ A third *Skil* KO mouse was generated by deleting the *Skil* gene promoter, resulting in alterations in T-cell activation.^179^ The knock-in mouse, expressing a mutant SnoN protein that lacks the ability to interact with Smads, showed a phenotype with partial embryo lethality due to defects in vascular system development. Studies using this transgenic mouse revealed a relevant function of SnoN to promote angiogenesis since SnoN interacts with the ALK1 receptor to increase the TGF-β/BMP9-dependent activation of Smad1 and Smad5.^180^ The expression of the SnoN protein is induced by TGF-β, activin, and nodal in human pluripotent embryonic stem cells, particularly to repress mesendodermal and primitive streak genes. Thus, the maintenance of embryonic stem-cell pluripotency is impaired by SnoN downregulation.^74^

Ski also exerts an important activity throughout embryonic development, specifically in formation of the central nervous system (CNS), skeletal muscle, and limbs.^30^ Endogenous Ski expression is increased during mouse embryonic development in several regions of the CNS; thus, *Ski*-KO mice show embryo lethality because closure of the cranial neural tube is impaired; there are also eye malformations, abnormalities in craniofacial structures, and a decrease in skeletal muscle mass. Likewise, *Ski*-knockdown (KD) mice show defects in eye and neural tube formation.^181^ *Ski*-KO mice mimic the same signs/symptoms of an anomaly named persistent hyperplastic primary vitreous (PHPV) observed in human and mouse models, which involves some ocular abnormalities, such as retinal malformations and microphtalmia.^182^ By contrast, overexpression of the Ski isoforms SkiA and SkiB gives rise to gastrulation alterations in zebrafish.^94^

### Neurogenesis

Ski and SnoN are important regulators during neurogenesis and for maintaining the homeostasis of the nervous system.^183–185^ Ski overexpression increases neural axis formation.^186^ Ski is needed for normal myelination of axons in the peripheral nervous system because it regulates Schwann cell proliferation and differentiation.^167^ In rats with a spinal cord injury, Ski is upregulated in reactive astrocytes, but not in neurons.^187^ The inhibition of Smad1 and Smad2 by Ski induces the expression of some neural markers;^188^ whereas, *Ski*-KO mice exhibit reduced expression of nestin, an intermediate filament protein of neuroepithelial cells and myocytes precursors;^189,190^ embryos of *Ski*-KO mice also exhibit extra vestigial-digits.^190^ Ski appears to maintain the identity of callosal neurons in the developing neurocortex by blocking *Ctip2* gene expression through a Ski/Stab2/HDAC1 complex.^65,66^ By contrast, SnoN modulates neuronal branching and positioning during axonal growth and regeneration (Fig. 6).^79,191,192^

### Hematopoiesis

Ski and SnoN are important regulators of cell differentiation; thus, they become key factors in the regulation of hematopoiesis.^193^ Ski induces the expression of genes involved in myeloid differentiation.^194^ Ski expression has been observed in megakaryocyte/erythrocyte dual-lineage progenitors, as well as in mature macrophages, mast cells, and B- and T-lineage cells.^193^ Ski mainly inhibits erythroid differentiation by inhibiting GATA1 activity or the RA signaling pathway in hematopoietic cells.^67,195^ Phorbol 12-myristate 3-acetate (PMA) can enhance Ski expression in human megakaryoblastic CHRF-288-11 cells that specifically undergo megakaryocytic differentiation;^196^ whereas, the process of megakaryopoiesis is stimulated by PMA, which favors activin A/Smad signaling and SnoN protein degradation (Fig. 6).^197^

### Myogenesis

Ski promotes muscular development by enhancing the expression of muscle regulatory factors; for instance, Ski overexpression in myoblasts induces expression of genes encoding muscle creatine kinase (*Mck*) and myosin light chain (*Mlc*).^198^ High Ski mRNA levels are observed during the late stages of muscle differentiation of mouse embryos and in cell lines.^199,200^ Overexpression of v-Ski induces *MyoD* and *myogenin* expression and promotes myogenesis.^87,201–203^ Ski-overexpressing transgenic mice exhibit an increased skeletal muscular mass;^204,205^ these mice exhibit high Ski mRNA levels in hind leg muscles.^206^ By contrast, transgenic cattle overexpressing chicken *Ski* also develop muscular hypertrophy, but later show muscle degeneration.^207^ Ski transgene overexpression in mice produces skeletal muscle hypertrophy; whereas, Ski overexpression in osteocytes causes skeletal abnormalities.^205,206^ Furthermore, Ski plays an important role in muscle regeneration; thus, higher Ski levels promote the proliferation of muscle cells.^208^ Other studies have shown an upregulation of Ski in some stages of axolotl embryogenesis, such as limb formation, as well as during axolotl limb regeneration.^92^ SnoN is involved in muscle cell differentiation by regulating the formation of muscle fibers in SnoN transgenic mice.^209^ TGF-β decreases *Ihh* mRNA levels in the neonatal growth plate via Smad2/SnoN and Ski/Smad3 complexes; this study shows that TGF-β signals antagonize *Ihh* expression, a key regulator of the proliferation and differentiation of chondrocytes (Fig. 6).^210^

### Metabolism

Ski is a key factor in the induction of myogenesis, but Ski may also decrease body fat mass by inducing oxidative metabolism of fatty acids. Transgenic mice overexpressing Ski show increased growth at early postnatal stages, as well as an altered body composition with decreased body fat and an enhanced lean body mass. The skeletal muscle of these transgenic mice has an enhanced fatty oxidative capacity and enhanced activity of both cytochrome C oxidase and citrate synthase; whereas, Ski represses some key genes in metabolism, such as *Srebp1* and *Pparγ*.^211^ Ski overexpression alters glycolysis and lactate production and enhances mitochondrial biogenesis and fatty acid oxidation; these effects may result after the upregulation of PPARγ. Ski overexpression also induces the expression of some PPARγ target genes that are implicated in lipid uptake, transport, and oxidation.^212^ Ski inhibits AKT phosphorylation induced by insulin in Ski-transgenic mice with resistance to diet-induced obesity.^213^

## Regulation of Tgf-β/smad signaling by Ski and Snon

### The role of Ski and SnoN in the TGF-β signaling network

The canonical TGF-β/Smad signaling pathway establishes a crosstalk with other pathways to efficiently achieve most of its biological functions, including the Wnt, Notch, Hippo, PI3K-AKT, PKC, MAPKs, and JAK-STAT signaling pathways. The regulation of Ski and SnoN protein expression is part of this crosstalk; thus, the TGF-β/R-Smad axis, activin, nodal, HGF/CREB/Sp1 axis, and prolactin/Stat5 axis are some of the known pathways that enhance SnoN expression in specific cell types (Fig. 2). Many other signals also control the SnoN protein levels, but the molecular mechanisms involved are unknown; for instance, certain EDCs increase SnoN in ovarian cancer cells, whereas oxymatrine and MG132 treatments induce SnoN in the damaged kidney of diabetic rats (Fig. 2).^115,214,215^ NFAT stabilizes SnoN to control TGF-β-induced EMT in MDA-MB-231 breast cancer cells.^216^ By contrast, other treatments are linked to SnoN downregulation; for example, HGF treatment decreases the SnoN protein levels in proliferating renal tubular epithelial HK2 cells.^217^ PMA induces activin A production, which activates Smad2 and Smad3 to promote SnoN degradation in myelogenous leukemia cells.^197^ Smurf2 and MAD2B proteins cause SnoN downregulation in the fibrotic kidney.^218,219^ The antibiotics anisomycin and puromycin induce Ski and SnoN degradation via UPS only in specific human cell types, such as A549 lung cancer cells, AD293 embryonic kidney cells, and A7 melanoma cells.^220–222^

TGF-β induces SnoN expression to inhibit the BMP2 and BMP7 signaling pathways in osteoblasts from patients undergoing total hip replacement.^223^ SnoN may also stabilize proteins, such as p53, TAZ, and STAT5. High levels of the SnoN/PML (promyelocytic leukemia) complex stabilize p53 and induce senescence in MEFs; whereas, the SnoN/p53 complex enhances p53-mediated transcription in MEFs.^45,224^ SnoN stabilizes TAZ protein by inhibiting its phosphorylation, which enhances TAZ/TEAD complex target gene expression during cell proliferation. SnoN cooperates with the Hippo pathway to induce transformation and promote EMT of breast cancer cells.^170^ SnoN is also induced by TGF-β and prolactin via Stat5 in late pregnancy; thus, SnoN blocks TGF-β signaling and stabilizes STAT5 protein to activate the prolactin pathway in lactogenesis.^112^

Ski also negatively controls the BMP, Hippo, Hedgehog, vitamin D, and RA signaling pathways. The Ski protein interacts with Lats2 and enhances its activity to destabilize the TAZ protein in mammary epithelial cells,^69^ whereas, in lung cancer, the *Ski* gene is silenced by methylation and, consequently, Ski is unable to inhibit TAZ.^101^ Ski and SnoN negatively regulate the expression of the *Indian hedgehog* (*Ihh*) gene in primary chondrocytes via different transcriptional complexes: SnoN/Smad2 and HDAC4 form a complex with high affinity; whereas, the Ski/Smad3 complex has a lower affinity.^210^ Furthermore, gefitinib is a drug that is used to treat lung cancer; thus, the IL-6 cytokine induces Stat3 phosphorylation to repress Smad3 expression, promoting gefitinib resistance, and Stat3 interacts with Ski and SnoN to inhibit the R-Smad/co-Smad complex.^225^

In summary, TGF-β participates in a crosstalk with many other pathways that may regulate the expression and/or abundance of the Ski and SnoN proteins, and concomitantly, Ski and SnoN exert diverse effects on the outcome of those signaling pathways through regulatory feedback loops that control the different cellular responses.

### The role of Ski and SnoN in the regulation of TGF-β signaling in disease

The physiological and pathological relevance of Ski and SnoN transcriptional cofactors has been clearly documented, but there is scarce information about their target genes and about the biological relevance of such gene regulation. Ski and SnoN may regulate gene expression in both Smad-dependent and -independent manners, and they may also depend on many other transcription factors to regulate gene transcription. This biological function of Ski and SnoN is important in tissue homeostasis, whereas alterations in Ski and SnoN expression and function have been linked to some diseases.

#### Wound healing and fibrosis

TGF-β superfamily members exert key roles during wound healing and fibrosis, regulating processes, such as myofibroblast (MFB) differentiation and EMT promotion.^226–228^ Ski and SnoN also exert differential effects during wound healing and tissue remodeling, depending on the cell context (Fig. 6). A few examples include the following: Ski and SnoN expression is enhanced during liver regeneration, probably to neutralize TGF-β/Smad antiproliferative actions;^171,229^ the regeneration of tendon-to-bone insertion is promoted by both SnoN overexpression and *Tgif* gene induction;^230^ Ski inhibits Smad3-induced apoptosis to promote cell proliferation in skin fibroblasts during wound healing;^231^ and Ski reduces scarring when it is used for gene therapy in rats.^232^ Ski also inhibits Smad3 activation but stimulates the p38 pathway to block TGF-β-induced vascular smooth muscle cell proliferation after vascular injury.^233^

Ski and SnoN cofactors may also mediate antifibrotic responses by blocking TGF-β signals. The renal model of fibrosis induced by unilateral urethral obstruction (UUO) exhibits an activated TGF-β pathway as a consequence of increased expression of Smurf2 and proteosomal degradation of the Ski and SnoN proteins.^150^ TGF-β profibrotic activity is inhibited after Ski and SnoN overexpression in renal cells, whereas the absence of SnoN sensitizes tubular epithelial HKC cells to TGF-β signaling.^234^ Smurf2 overexpression promotes SnoN downregulation in renal proximal tubule epithelial cells when they are cultured under high-glucose conditions;^218^ whereas, SnoN overexpression protects these tubular epithelial cells from undergoing EMT.^235^ Furthermore, the alkaloid oxymatrine extracted from the herb *Sephora japonica* prevents SnoN downregulation in renal tubules during the EMT process induced by high glucose.^215^ By contrast, enhancement of SnoN protein stability due to treatment with MG132 decreases kidney damage in diabetic rats.^214^ Likewise, Smurf2 knockdown increases SnoN levels and blocks TGF-β signaling in rats with obstructive nephropathy.^236^ It is clear that the polyubiquitination and degradation of SnoN via Smurf2 induced by the TGF-β/Smad pathway is critical in diabetic nephropathy.^237^ BMP7 enhances the levels of SnoN mRNA and protein, which is important for controlling diabetic nephropathy and renal fibrosis.^113^ Moreover, the upregulation of SnoN inhibits EMT and renal fibrogenesis induced by high glucose. The miR-23a causes SnoN downregulation, whereas depletion of miR-23a increases the SnoN protein levels and decreases EMT and renal fibrogenesis.^117^ Interestingly, the expression of SnoN, TGF-β, and Arkadia can be increased in renal tubular cells treated with high glucose, whereas the SnoN protein levels are decreased; however, the SnoN protein levels can be increased if Arkadia is downregulated, leading to the inhibition of high glucose-induced EMT.^238^ Similarly, fibrogenesis in different tissues correlates with changes in SnoN levels, as occurs in the fibrotic lung^239^ and during renal fibrosis.^219^ Thus, regulation of SnoN protein abundance seems to be key in the implementation of therapeutic strategies for diabetic nephropathy.

TGF-β induces cellular matrix production by activating MFB during cardiac fibrogenesis; however, transient Ski overexpression in MFB decreases Smad2 phosphorylation, which results in a decrease in type I collagen synthesis and α-SMA levels.^169,240^ In this case, stable overexpression of Ski promotes cardiac MFB apoptosis.^241^ Bone marrow progenitor cell therapy modulates cardiac fibrosis in diabetic hearts by decreasing miR-155 levels, whereas overexpression of miR-155 in cardiac fibroblasts inhibits Ski and SnoN expression.^242^ Ski overexpression also induces *Meox2* gene expression and blocks *Zeb2* gene expression, affecting the cardiac MFB phenotype.^243^ Furthermore, TGF-β can induce endothelial-mesenchymal transition (EndMT) of human coronary artery endothelial cells (HCAECs) to generate matrix-producing fibroblasts and promote cardiac fibrosis. Importantly, Ski overexpression inhibits the TGF-β-induced EndMT of HCAECs via a mechanism that involves the regulation of miR-155.^107^ During liver fibrogenesis induced by TGF-β and acetaldehyde, an ethanol metabolite, increased collagen synthesis occurs in hepatic stellate cells (HSC); in these cells, the Ski/Smad4 complex is translocated to the cytoplasm, and the Ski protein is degraded via UPS.^244^ In systemic sclerosis, the levels of the Ski and SnoN proteins are increased in scleroderma fibroblasts, although they fail to inhibit the TGF-β pathway because they are unable to compete with the p300 coactivator.^245^ Regarding dermal tissues, Ski transgenic overexpression promotes wound healing in rat dermal wounds and inhibits scar formation in rabbit ears.^246^

#### Carcinogenesis

The TGF-β cytokine exerts tumor-suppressive actions that include inhibition of cellular proliferation and immortalization, and it also promotes apoptosis in normal cells and early carcinomas. By contrast, the tumor-promoting effects of TGF-β include the promotion of EMT, cell migration, invasion, and metastasis.^247–249^ Thus, TGF-β cytostatic and protective effects are frequently lost as tumors develop. Loss-of-TGF-β signaling is involved in hyperproliferative disorders, inflammation, autoimmune diseases, and tumor formation; whereas, gain of TGF-β signaling promotes immunosuppression and tumor metastasis.^3^ In addition, most tumors may arise after mutations or deletions in genes encoding components of the TGF-β signaling pathway.^3,250^

Ski and SnoN were initially described as proto-oncoproteins because of their ability to induce cellular transformation in vitro.^50,89,201,202,251,252^ Interestingly, the Ski and SnoN heterodimers are better able to induce cellular transformation than their respective homodimers or monomers.^50,130^ Thus, SnoN and Ski are up- or downregulated in many types of cancer cells, including leukemia, lymphoma, melanoma, breast cancer, cervical cancer, esophageal squamous-cell carcinoma, colorectal carcinoma, pancreatic cancer, and gastrointestinal tumors.^85,104,178,253–272^ However, it is worth mentioning that no mutations of Ski and SnoN have been found in any of the different cancer types studied to date.

Currently, it has been observed that Ski and SnoN are differentially expressed in normal and cancerous cells, and some evidence also supports the alteration of their localization, abundance, and function in cancer. *Skil* heterozygous mice develop spontaneous lymphoma and are quite sensitive to carcinogens, revealing the anti-oncogenic activity of SnoN. The T-cells, B-cells, and fibroblasts of these mice exhibit resistance to apoptosis and cell cycle arrest, supporting the role of SnoN as a tumor suppressor.^178^ Likewise, Ski tumor suppression activity has been observed in Ski-deficient mice, which exhibit higher sensitivity to tumor formation induced with carcinogens.^273^ Ski loss increases MEF proliferation, whereas Ski overexpression inhibits MEF proliferation via transcriptional gene repression in association with Rb and MAD.^273^ Both SnoN and Ski seem to be important in some cancer types. A correlation between β-catenin activation and upregulation of the SnoN and Ski proteins has been observed in early stages of colorectal cancer.^262^ Another study has shown that the p53 protein is stabilized after the SnoN protein is recruited to PML nuclear bodies through PML, which induces cellular senescence in a Smad-independent manner and inhibits tumorigenesis;^45,224^ by contrast, Ski inhibits p53 activity and promotes p53 protein degradation.^68,156^ Defective control of TGF-β signaling is responsible for cancer induction in Barret´s esophagus; patients with low-grade dysplasia exhibit low Ski and SnoN protein levels in dysplasic areas, whereas these proteins are absent in patients with high-grade dysplasia/adenocarcinoma.^274^

The mechanisms linked to SnoN deregulation in cancer have been better studied. Altered *Skil* gene transcriptional regulation, *Skil* gene amplification, or higher SnoN protein stability are among the main causes of SnoN upregulation, whereas SnoN can be decreased by allelic loss in some cancer types. Hence, SnoN expression in human colorectal cancer is downregulated in <40% of tumors with high-level microsatellite instability, whereas 50% of microsatellite-stable tumors present an upregulation of SnoN.^275^ Furthermore, in 179 human colorectal tumor biopsies, 55.2% of tumors have either partial or complete allelic loss of the *Skil* gene, whereas 15.1% of tumors present amplification of the *Skil* gene.^256^ *Skil* gene amplification was initially observed in esophagus squamous cell carcinoma,^254^ but it has also been observed in immortalized human mammary epithelial cells, where *Skil* and *Tloc1* may cooperate to promote cell proliferation, transformation, invasion, and tumor growth.^276^ By contrast, TGF-β is unable to induce SnoN protein degradation in human esophageal cancer cell lines and in rat AS-30D hepatoma cells; indeed, high levels of the SnoN protein have been detected in both cancer types.^172,260^ *Skil* and *Ski* genes are often co-amplified with other genes with oncogenic activity. For instance, *Skil and Tloc1* genes are contained in the 3q26 chromosome, specifically in a region that is frequently amplified in several cancer types.^276^ The progression of nasopharyngeal carcinoma is associated with co-amplification of *Gpr160* and *Skil* genes, both of which are localized at 3q26.2-q26.32, along with deletion of the *AdamtS9* and *Lrig1* genes, which are localized at 3p12.3-p14.2.^277^ The 3q26 region is often amplified in ovarian cancer, increasing the mRNA levels of some genes, such as *Skil*, *Evi1*, and *Plscr1*.^81,278,279^ The *Ski* gene is co-amplified with the *Mel1* gene in MKN28 gastric cancer cells; both genes are localized at 1p36 and cooperate to inhibit TGF-β anti-proliferative actions.^267^

#### Metastasis

The role of Ski in cancer metastasis depends on the context and cellular type.^273^ The Ski protein levels are decreased in metastatic non-small lung cancer cells (NSCLCs) and lung cancer tissues, whereas Ski overexpression blocks the TGF-β-mediated EMT process.^280^ Ski downregulation by RNAi inhibits human melanoma growth in vivo,^264^ whereas it has a dual effect on pancreatic-cancer tumorigenesis because *Ski*-KD inhibits tumor growth and enhances metastasis to the lung; additionally, Ski downregulation alters the expression of TGF-β target genes linked to pancreatic cancer cell metastasis.^268^ Ski also participates in maintaining the stemness of pancreatic cancer stem cells by promoting the expression of components of the sonic hedgehog (Shh) pathway, such as Shh, Ptch-1, Smo, Gli-1, and Gli-2.^281^ Ski overexpression has been observed in acute myeloid leukemia (AML), where Ski inhibits retinoic acid receptor (RAR) signaling.^282^ Ski upregulation activates cancer-associated fibroblasts (CAFs) in breast tumors to promote breast cancer cell invasion.^283^ In addition, the expression of HPV16 genes is mediated by NF1 in association with Ski in cervical carcinoma.^85^ Ski is a negative prognostic marker in primary esophageal squamous carcinoma,^257^ pancreatic ductal adenocarcinoma (PDAC),^268^ colorectal carcinoma,^256^ and gastric cancer.^269^ Untreated patients with chronic lymphocytic leukemia (CLL) have a two-gene signature consisting of *Ski* and *Slamf1* that predicts time to treatment and survival.^284^ High levels of cytoplasmic Ski negatively correlate with tumor size, stage, and lymph node status in invasive breast carcinomas and positively with survival.^285^ Interestingly, the *Ski* gene is methylated in lung cancer, which correlates with the progression of this type of cancer. Under this scenario, Ski fails to block TAZ activity and the proliferation of lung cancer cells.^101^ Moreover, one of the treatments to regulate Ski protein levels includes the use of the TGF-β inhibitory P144 peptide, which is derived from the extracellular sequence of the human TGF-β type III receptor, and interestingly, P144 promotes Ski downregulation in human glioblastoma cell lines, such as A172 and U-87 MG.^286^

SnoN duality as an oncoprotein and tumor suppressor was confirmed through xenotransplantation in athymic mice of lung A549 and breast MDA-MB-231 cancer cells expressing either high or low SnoN protein levels. Surprisingly, xenotransplant of breast cancer cells expressing low SnoN levels exhibited high cell metastasis to the bones and lungs, but poor tumor development.^287^ SnoN is an important regulator of EMT induced by TGF-β in MDA-MB231 breast cancer cells. During EMT, upregulated NFAT modifies SnoN subcellular localization from the cytoplasm to the nucleus and prevents SnoN degradation by sequestering Smad3. NFAT cooperates with SnoN to promote TGF-β-induced EMT by increasing the expression of genes encoding MMP-2, MMP-9, and N-cadherin.^216^ Moreover, sumoylation of SnoN by PIAS1 has an inhibitory effect on the invasive capacity of MDA-MB231 cells.^288^ Particularly for SnoN, it has been reported that this protein regulates cell differentiation in normal skin and in benign skin tumors, but it promotes squamous cell carcinoma.^289^ SnoN induces proliferation of ovarian cancer cells, whereas it promotes cell cycle arrest and senescence in non-transformed ovarian epithelial cells.^279^ SnoN oncogenic activity can induce cellular proliferation and inhibit apoptosis of other cancer cells, such as pancreatic cancer cells^272^ (Table 1).

**Table 1:** Ski and SnoN levels in cancer

Cancer types	Cofactor	Expression	Mechanism	References
Acute myeloid leukemia	Ski	Upregulated	Loss-of-Ski regulation by miR-29a	^104,282^
Breast cancer	SnoN	Upregulated	N.D.	^255^
Cervical	Ski	Upregulated	N.D.	^85,271^
Chronic Myelogenus Leukemia	Ski	Upregulated	N.D.	^258^
Colorectal	Ski	Downregulated	Partial or complete allelic loss	^256,262^
		Upregulated	*Ski* gene amplification	
	SnoN	Downregulated	Partial or complete allelic loss	^256,275^
		Upregulated	*Skil* gene amplification	
Esophageal	Ski	Upregulated	N.D.	^257,274^
	SnoN	Upregulated	Stability of SnoN protein	^254,260,274^
			*Skil* gene amplification	
Gastrointestinal	Ski/SnoN	Upregulated	N.D.	^265,267,269,270^
Hemangioma	Ski	Upregulated	N.D.	^266^
Hepatocarcinoma	SnoN/Ski	Upregulated	N.D.	^172^
Lymphoma	SnoN	Downregulated	*sno-*heterozygous mice	^178^
Lung cancer	Ski	Downregulated	*Ski* gene methylation	^101^
Melanoma	Ski/SnoN	Upregulated	N.D.	^162,163,253,259,263,264^
Nasopharyngeal	SnoN	Upregulated	*Skil* gene amplification	^277^
Ovarian	SnoN	Upregulated	*Skil* gene amplification	^81,279^
Pancreatic	Ski/SnoN	Upregulated	N.D.	^261,268,272,281^

*N.D.* Not Demonstrated

#### Human genetic diseases

Shprintzen-Goldberg syndrome (SGS) is a human disorder characterized by craniofacial, cardiovascular, neuromuscular, and skeletal anomalies.^290,291^ The specific mutation consists of an in-frame deletion in exon 1 of the human *Ski* gene; this mutation falls within the Ski protein region of interaction with R-Smad.^290–292^ Thus, SGS alterations correlate with uncontrolled TGF-β signaling activation since mutant Ski cannot bind Smad proteins. Analysis of the mutation frequency in patients with SGS demonstrates that the Smad-binding domain of Ski is a hot spot for multiple de novo mutations.^291,293^ Intriguingly, *Ski*-KO mouse and *Ski*-KD zebrafish have similar phenotypes to those observed in SGS, i.e., craniofacial abnormalities and aortic aneurysms, among others (Fig. 6).^220,293^

### Ski and SnoN as targets in therapies to regulate TGF-β signaling

The TGF-β signaling pathway is deregulated in different diseases, such as fibrosis and cancer. Multiple pharmacological compounds have been used to target the components of the TGF-β pathway, such as anti-sense oligonucleotides, antibodies, and kinase inhibitors, among others. However, it has been a real challenge to target the components of this pathway due to its pleiotropic nature and because of the side effects generated by the pharmacological agents.^294^ Furthermore, the applicability of TGF-β pathway inhibitors for therapeutic treatments must be carefully considered since TGF-β signaling blockade has been demonstrated to cause an upregulation of Ski and SnoN as a result of the loss of stability regulation of these proteins exerted by Smads.

Like TGF-β signaling, Ski and SnoN also participate in the homeostasis of many physiological processes; consequently, the deregulation of these cofactors is associated with disease development, such as fibrosis and cancer. Fibrosis of some organs, such as the kidney, lung, and liver, seems to be linked to the reduction of Ski and SnoN levels and activation of the TGF-β pathway.^218,219,234,236,239^ The treatments for fibrosis and associated diseases, such as diabetic nephropathy, will benefit from strategies to upregulate Ski and SnoN expression to antagonize TGF-β pro-fibrotic actions.^235^ For instance, it has been suggested that Ski and SnoN accumulation after treatment with proteasome inhibitors (such as MG132) is associated with the prevention of fibrotic damage in the kidney of diabetic rats.^214^ In addition, the use of alkaloids, such as oxymatrine, blocks SnoN downregulation in a cellular model of kidney tubulo-interstitial fibrosis,^215^ whereas treatment with omega-3 fatty acid increases SnoN expression in fibrotic lungs.^239^ Regarding tissue regeneration, it has been hypothesized that the upregulation of Ski and SnoN may promote liver regeneration by antagonizing the antiproliferative actions of TGF-β;^171,172,229^ likewise, the upregulation of SnoN stimulates axonal growth and regeneration.^79,191,192^

In carcinogenesis, Ski and SnoN act as oncoproteins when overexpressed; SnoN can be upregulated by gene amplification, elevated protein stability, or by enhanced *Skil* gene transcription, whereas SnoN can be downregulated by partial or complete allelic loss of the *Skil* gene. Ski and SnoN levels are also upregulated in some cancer types, where some of their main regulators, such as Smad2/3/4 or E3 ubiquitin ligases, are mutated or non-functional.^295^ By contrast, SnoN expression is increased, whereas Ski expression is downregulated, after *Ski* gene methylation in lung cancer. Therefore, the design of strategies focused on the downregulation of these cofactors might be useful to control tumor growth. For instance, the downregulation of SnoN with specific interfering RNAs reduces proliferation and promotes apoptosis of hepatoma and pancreatic cancer cells.^272,296^ Of note, there is a pitfall associated with a significant reduction of Ski or SnoN levels since this approach can lead to the promotion of tumor metastasis, as has been shown in breast, lung, and pancreatic cancer cells, and thus the use of this strategy as an anti-cancer therapy can result in a serious disadvantage.^268,287^

## Concluding remarks

The Ski and SnoN proteins play critical roles in health and disease by controlling the outcome of TGF-β and other signaling pathways.^28–30,33,297^ Therefore, the maintenance of balanced Ski and SnoN expression levels must be tightly controlled to normalize the TGF-β signaling outcome in diverse pathologies. Therefore, Ski and SnoN are potential therapeutic targets under the pathological conditions in which they are deregulated. The design of therapeutic strategies must be focused on restoring the expression levels of Ski and SnoN, with the purpose of recovering the function of TGF-β signaling and, perhaps, other signaling pathways, as well as re-establishing cellular homeostasis. Notably, the Ski and SnoN proteins are downregulated in fibrosis and cancer metastasis and upregulated in tumor growth; thus, it is important to develop diverse therapeutic strategies to target Ski and SnoN to regulate the TGF-β signaling outcome by attacking only the pro-fibrotic and tumor-promoting effects of this cytokine.

## References

[1] Hinck AP, Mueller TD, Springer TA (2016). Structural biology and evolution of the TGF-β family. Cold Spring Harb. Perspect. Biol..

[2] Moses, H. L., Roberts, A. B. The discovery of TGF-β: A historical perspective. In: Derynck R., Miyazono K. (eds). *The TGF-β Family*, vol. 50. Cold Spring Harbor Laboratory Press: New York, 2008, pp 1–28.

[3] Massagué J, Blain SW, Lo RS (2000). TGFβ signaling in growth control, cancer, and heritable disorders. Cell.

[4] Wrana, J. L., Ozdamar, B., Le Roy, C., Benchabane, H. Signaling Receptors of the TGF β family. In: Derynck R., Miyazono K. (eds). *The TGF-β Family*, vol. 50. Cold Spring Harbor Laboratory Press: New York, 2008, pp 151–178.

[5] Heldin CH, Moustakas A (2016). Signaling receptors for TGF-β family members. Cold Spring Harb. Perspect. Biol..

[6] Wrana JL, Attisano L, Wieser R, Ventura F, Massagué J (1994). Mechanism of activation of the TGF-β receptor. Nature.

[7] Wieser R, Wrana JL, Massagué J (1995). GS domain mutations that constitutively activate T beta R-I, the downstream signaling component in the TGF-beta receptor complex. EMBO J..

[8] Raftery LA, Twombly V, Wharton K, Gelbart WM (1995). Genetic screens to identify elements of the decapentaplegic signaling pathway in Drosophila. Genetics.

[9] Hoodless PA (1996). MADR1, a MAD-related protein that functions in BMP2 signaling pathways. Cell.

[10] Savage C (1996). Caenorhabditis elegans genes sma-2, sma-3, and sma-4 define a conserved family of transforming growth factor beta pathway components. Proc. Natl Acad. Sci. USA.

[11] Macías-Silva M (1996). MADR2 is a substrate of the TGF-β receptor and its phosphorylation is required for nuclear accumulation and signaling. Cell.

[12] Lagna G, Hata A, Hemmati-Brivanlou A, Massagué J (1996). Partnership between DPC4 and SMAD proteins in TGF-beta signalling pathways. Nature.

[13] Abdollah S (1997). TbetaRI phosphorylation of SMAD2 on Ser^465^ and Ser^467^ is required for SMAD2-SMAD4 complex formation and signaling. J. Biol. Chem..

[14] Souchelnytskyi S (1997). Phosphorylation of Ser^465^ and Ser^467^ in the C terminus of SMAD2 mediates interaction with SMAD4 and is required for transforming growth factor-beta signaling. J. Biol. Chem..

[15] Liu X (1997). Transforming growth factor beta-induced phosphorylation of SMAD3 is required for growth inhibition and transcriptional induction in epithelial cells. Proc. Natl Acad. Sci. USA.

[16] Kretzschmar M, Liu F, Hata A, Doody J, Massagué J (1997). The TGF-beta family mediator SMAD1 is phosphorylated directly and activated functionally by the BMP receptor kinase. Genes Dev..

[17] Macías-Silva M, Hoodless PA, Tang SJ, Buchwald M, Wrana JL (1998). Specific activation of SMAD1 signaling pathways by the BMP7 type I receptor, ALK2. J. Biol. Chem..

[18] Wrana JL (2002). Phosphoserine-dependent regulation of protein-protein interactions in the SMAD pathway. Structure.

[19] Lin X, Chen G, Feng H, Derynck R, Miyazono K (2008). Transcriptional control via SMADs. The TGF-β Family.

[20] Gaarenstroom T, Hill CS (2014). TGF-β signaling to chromatin: How SMADs regulate transcription during self-renewal and differentiation. Semin. Cell Dev. Biol..

[21] Barrios-Rodiles M (2005). High-throughput mapping of a dynamic signaling network in mammalian cells. Science.

[22] Aragon E (2011). A SMAD action turnover switch operated by WW domain readers of a phosphoserine code. Genes Dev..

[23] Macias MJ, Martin-Malpartida P, Massagué J (2015). Structural determinants of SMAD function in TGF-β signaling. Trends Biochem. Sci..

[24] Xu P, Lin X, Feng XH (2016). Posttranslational regulation of SMADs. Cold Spring Harb. Perspect. Biol..

[25] Miyazono K (2000). TGF-beta signaling by SMAD proteins. Cytokine Growth Factor Rev..

[26] Vizan P (2013). Controlling long-term signaling: receptor dynamics determine attenuation and refractory behavior of the TGF-β pathway. Sci. Signal..

[27] Stroschein SL, Wang W, Zhou S, Zhou Q, Luo K (1999). Negative feedback regulation of TGF-beta signaling by the SnoN oncoprotein. Science.

[28] Liu X, Sun Y, Weinberg RA, Lodish HF (2001). Ski/Sno and TGF-β signaling. Cytokine Growth Factor Rev..

[29] Luo K (2004). Ski and SnoN: negative regulators of TGF-β signaling. Curr. Opin. Genet. Dev..

[30] Bonnon C, Atanasoski S (2012). c-Ski in health and disease. Cell Tissue Res..

[31] Stavnezer E, Barkas AE, Brennan LA, Brodeur D, Li Y (1986). Transforming Sloan-Kettering viruses generated from the cloned v-ski oncogene by in vitro and in vivo recombinations. J. Virol..

[32] Sutrave P, Copeland TD, Showalter SD, Hughes SH (1990). Characterization of chicken c-ski oncogene products expressed by retrovirus vectors. Mol. Cell Biol..

[33] Deheuninck J, Luo K (2009). Ski and SnoN, potent negative regulators of TGF-β signaling. Cell Res..

[34] Mardon G, Solomon NM, Rubin GM (1994). Dachshund encodes a nuclear protein required for normal eye and leg development in Drosophila. Development.

[35] Hammond KL, Hanson IM, Brown AG, Lettice LA, Hill RE (1998). Mammalian and Drosophila dachshund genes are related to the Ski proto-oncogene and are expressed in eye and limb. Mech. Dev..

[36] Kozmik Z (1999). Molecular cloning and expression of the human and mouse homologues of the Drosophila dachshund gene. Dev. Genes. Evol..

[37] Caubit X (1999). Mouse Dac, a novel nuclear factor with homology to Drosophila dachshund shows a dynamic expression in the neural crest, the eye, the neocortex, and the limb bud. Dev. Dyn..

[38] Davis RJ, Shen W, Heanue TA, Mardon G (1999). Mouse Dach, a homologue of Drosophila dachshund, is expressed in the developing retina, brain and limbs. Dev. Genes Evol..

[39] Davis RJ, Shen W, Sandler YI, Heanue TA, Mardon G (2001). Characterization of mouse Dach2, a homologue of Drosophila dachshund. Mech. Dev..

[40] Da Graca LS (2004). DAF-5 is a Ski oncoprotein homolog that functions in a neuronal TGFβ pathway to regulate C. elegans dauer development. Development.

[41] Tewari M (2004). Systematic interactome mapping and genetic perturbation analysis of a C. elegans TGF-β signaling network. Mol. Cell.

[42] Tavsanli BC (2004). Structure-function analysis of the Drosophila retinal determination protein Dachshund. Dev. Biol..

[43] Arndt S, Poser I, Schubert T, Moser M, Bosserhoff AK (2005). Cloning and functional characterization of a new Ski homolog, Fussel-18, specifically expressed in neuronal tissues. Lab. Invest..

[44] Arndt S, Poser I, Moser M, Bosserhoff AK (2007). Fussel-15, a novel Ski/Sno homolog protein, antagonizes BMP signaling. Mol. Cell Neurosci..

[45] Pan D, Zhu Q, Luo K (2009). SnoN functions as a tumour suppressor by inducing premature senescence. EMBO J..

[46] Arndt S, Schmidt J, Wacker E, Karrer S, Bosserhoff AK (2011). Fussel-15, a new player in wound healing, is deregulated in keloid and localized scleroderma. Am. J. Pathol..

[47] Fischer S (2012). Fussel (fuss)-A negative regulator of BMP signaling in Drosophila melanogaster. PLoS ONE.

[48] Takaesu NT (2012). Drosophila CORL is required for SMAD2-mediated activation of Ecdysone Receptor expression in the mushroom body. Development.

[49] Nicol R, Stavnezer E (1998). Transcriptional repression by v-Ski and c-Ski mediated by a specific DNA binding site. J. Biol. Chem..

[50] Nicol R, Zheng G, Sutrave P, Foster DN, Stavnezer E (1999). Association of specific DNA binding and transcriptional repression with the transforming and myogenic activities of c-Ski. Cell Growth Differ..

[51] Luo K (1999). The Ski oncoprotein interacts with the SMAD proteins to repress TGFβ signaling. Genes Dev..

[52] Sun Y (1999). Interaction of the Ski oncoprotein with SMAD3 regulates TGF- β signaling. Mol. Cell.

[53] Xu W (2000). Ski acts as a co-repressor with SMAD2 and SMAD3 to regulate the response to type β transforming growth factor. Proc. Natl Acad. Sci. USA.

[54] Tabata T, Kokura K, ten Dijke P, Ishii S (2009). Ski co-repressor complexes maintain the basal repressed state of the TGF-β target gene, SMAD7, via HDAC3 and PRMT5. Genes Cell.

[55] Nagase T (1990). Requirement of protein co-factor for the DNA-binding function of the human ski proto-oncogene product. Nucleic Acids Res..

[56] Akiyoshi S (1999). c-Ski acts as a transcriptional co-repressor in transforming growth factor-β signaling through interaction with SMADs. J. Biol. Chem..

[57] Dai P (2002). Ski is involved in transcriptional regulation by the repressor and full-length forms of Gli3. Genes Dev..

[58] Ueki N, Hayman MJ (2003). Direct interaction of Ski with either SMAD3 or SMAD4 is necessary and sufficient for Ski-mediated repression of transforming growth factor-beta signaling. J. Biol. Chem..

[59] Chen W (2007). Competition between Ski and CREB-binding protein for binding to SMAD proteins in transforming growth factor- β signaling. J. Biol. Chem..

[60] Harada J (2003). Requirement of the co-repressor homeodomain-interacting protein kinase 2 for ski-mediated inhibition of bone morphogenetic protein-induced transcriptional activation. J. Biol. Chem..

[61] Pessah M (2002). C-Jun associate with the oncoprotein Ski and suppresses SMAD2 transcriptional activity. J. Biol. Chem..

[62] Ueki N, Hayman MJ (2003). Signal-dependent N-CoR requirement for repression by the Ski oncoprotein. J. Biol. Chem..

[63] Ueki N, Zhang L, Hayman MJ (2008). Ski can negatively regulates macrophage differentiation through its interaction with PU.1. Oncogene.

[64] Zhao HL, Ueki N, Marcelain K, Hayman MJ (2009). The Ski protein can inhibit ligand induced RARα and HDAC3 degradation in the retinoic acid signaling pathway. Biochem. Biophys. Res. Commun..

[65] Baranek C, Atanasoski S (2012). Modulating epigenetic mechanisms: the diverse functions of Ski during cortical development. Epigenetics.

[66] Baranek C (2012). Protooncogene Ski cooperates with the chromatin-remodeling factor Satb2 in specifying callosal neurons. Proc. Natl Acad. Sci. USA.

[67] Ueki N, Zhang L, Hayman MJ (2004). Ski negatively regulates erythroid differentiation through its interaction with GATA1. Mol. Cell Biol..

[68] Inoue Y, Iemura S, Natsume T, Miyazawa K, Imamura T (2011). Suppression of p53 activity through the cooperative action of Ski and histone deacetylase SIRT1. J. Biol. Chem..

[69] Rashidian J (2015). Ski regulates Hippo and TAZ signaling to suppress breast cancer progression. Sci. Signal..

[70] Tokitou F (1999). Viral ski inhibits retinoblastoma protein (Rb)-mediated transcriptional repression in a dominant negative fashion. J. Biol. Chem..

[71] Nomura T (1999). Ski is a component of the histone deacetylase complex required for transcriptional repression by Mad and thyroid hormone receptor. Genes Dev..

[72] Kokura K (2001). The Ski protein family is required for MeCP2-mediated transcriptional repression. J. Biol. Chem..

[73] Tecalco-Cruz AC (2012). Transforming growth factor-β/SMAD Target gene SKIL is negatively regulated by the transcriptional cofactor complex SNON-SMAD4. J. Biol. Chem..

[74] Tsuneyoshi N (2012). The SMAD2/3 corepressor SNON maintains pluripotency through selective repression of mesendodermal genes in human ES cells. Genes Dev..

[75] Briones-Orta MA, Sosa-Garrocho M, Moreno-Alvarez P, Fonseca-Sánchez MA, Macías-Silva M (2006). SnoN co-repressor binds and represses SMAD7 gene promoter. Biochem. Biophys. Res. Commun..

[76] Wilkinson DS, Tsai WW, Schumacher MA, Barton MC (2008). Chromatin-bound p53 anchors activated SMADs and the mSin3A corepressor to confer Transforming-Growth-Factor-β-mediated transcription repression. Mol. Cell Biol..

[77] Sarker KP, Wilson SM, Bonni S (2005). SnoN is a cell type-specific mediator of transforming growth factor-beta responses. J. Biol. Chem..

[78] Sarker KP (2008). ING2 as a novel mediator of transforming growth factor-β-dependent responses in epithelial cells. J. Biol. Chem..

[79] Ikeuchi Y (2009). A SnoN-Ccd1 pathway promotes axonal morphogenesis in the mammalian brain. J. Neurosci..

[80] Band AM, Laiho M (2012). SnoN oncoprotein enhances estrogen receptor-α transcriptional activity. Cell Signal..

[81] Kodigepalli KM, Anur P, Spellman P, Sims PJ, Nanjundan M (2013). Phospholipid Scramblase 1, an interferon-regulated gene located at 3q23, is regulated by SnoN/SkiL in ovarian cancer cells. Mol. Cancer.

[82] Solomon E, Li H, Duhachek Muggy S, Syta E, Zolkiewska A (2010). The role of SnoN in transforming growth factor beta1-induced expression of metalloprotease-disintegrin ADAM12. J. Biol. Chem..

[83] Shinozuka E (2013). SnoN/SKIL modulates proliferation through control of hsa-miR-720 transcription in esophageal cancer cells. Biochem. Biophys. Res. Commun..

[84] Tarapore P (1997). DNA binding and transcriptional activation by the Ski oncoprotein mediated by interaction with NFI. Nucleic Acids Res..

[85] Baldwin A, Pirisi L, Creek KE (2004). NFI-Ski interactions mediate Transforming Growth Factor β modulation of human papillomavirus type 16 early gene expression. J. Virol..

[86] Chen D (2003). SKI activates Wnt/beta-catenin signaling in human melanoma. Cancer Res..

[87] Kobayashi N (2007). c-Ski activates MyoD in the nucleus of myoblastic cells through suppression of histone deacetylases. Genes Cell.

[88] Zhang H, Stavnezer E (2009). Ski regulates muscle terminal differentiation by transcriptional activation of Myog in a complex with Six1 and Eya3. J. Biol. Chem..

[89] Stavnezer E, Gerhard DS, Binari RC, Balazs I (1981). Generation of transforming viruses in cultures of chicken fibroblasts infected with an avian leukosis virus. J. Virol..

[90] Li Y, Turck CM, Teumer JK, Stavnezer E (1986). Unique sequence, ski, in Sloan-Kettering avian retroviruses with properties of a new cell-derived oncogene. J. Virol..

[91] Nomura N (1989). Isolation of human cDNA clones of ski and the ski-related gene, sno. Nucleic Acids Res..

[92] Ludolph DC (1995). Cloning and expression of the axolotl proto-oncogene ski. Biochim. Biophys. Acta.

[93] Huang CJ, Lin JY, Tsai HJ (1999). Two distinct c-ski cDNAs of fish, tilapia (Oreochromis aurea). Mol. Reprod. Dev..

[94] Kaufman CD, Martínez-Rodríguez G, Hackett PB (2000). Ectopic expression of c-ski disrupts gastrulation and neural patterning in zebrafish. Mech. Dev..

[95] Grimes HL, Szente BE, Goodenow MM (1992). C-ski cDNAs are encoded by eight exons, six of which are closely linked within the chicken genome. Nucleic Acids Res..

[96] Grimes HL, Ambrose MR, Goodenow MM (1993). C-ski transcripts with and without exon 2 are expressed in skeletal muscle and throughout chick embryogenesis. Oncogene.

[97] Leferovich JM, Lana DP, Sutrave P, Hughes SH, Kelly AM (1995). Regulation of c-ski transgene expression in developing and mature mice. J. Neurosci..

[98] Zhang SX (2005). Identification of direct serum-response factor gene targets during Me2SO-induced P19 cardiac cell differentiation. J. Biol. Chem..

[99] Li J (2012). Upregulation of ski in fibroblast is implicated in the peroxisome proliferator-activated receptor δ-mediated wound healing. Cell Physiol. Biochem..

[100] Melling MA, Friendship CR, Shepherd TG, Drysdale TA (2013). Expression of Ski can act as a negative feedback mechanism on retinoic acid signaling. Dev. Dyn..

[101] Xie M, Wu X, Zhang J, Li X (2017). Ski regulates Smads and TAZ signaling to suppress lung cancer progression. Mol. Carcinog..

[102] Imig J (2011). microRNA profiling in Epstein-Barr virus-associated B-cell lymphoma. Nucleic Acids Res..

[103] Levati L (2011). MicroRNA-155 targets the SKI gene in human melanoma cell lines. Pigment Cell Melanoma Res..

[104] Teichler S (2011). MicroRNA29a regulates the expression of the nuclear oncogene Ski. Blood.

[105] Jiang H (2014). Next generation sequencing analysis of miRNAs: MiR-127-3p inhibits glioblastoma proliferation and activates TGF-β signaling by targeting SKI. OMICS.

[106] Li J, Zhao L, He X, Yang T, Yang K (2014). MiR-21 inhibits c-Ski signaling to promote the proliferation of rat vascular smooth muscle cells. Cell Signal..

[107] Wang J (2017). The mechanism of TGF-β/miR-155/c-Ski regulates endothelial-mesenchymal transition in human coronary artery endothelial cells. Biosci. Rep..

[108] Zhu Q, Pearson-White S, Luo K (2005). Requirement for the SnoN oncoprotein in transforming growth factor β-induced oncogenic transformation of fibroblast cells. Mol. Cell Biol..

[109] Denissova NG, Liu F (2004). Repression of endogenous SMAD7 by Ski. J. Biol. Chem..

[110] Tan R, Zhang X, Yang J, Li Y, Liu Y (2007). Molecular basis for the cell type specific induction of SnoN expression by hepatocyte growth factor. J. Am. Soc. Nephrol..

[111] Mayoral R (2010). Impairment of transforming growth factor β signaling in caveolin-1-deficient hepatocytes: role in liver regeneration. J. Biol. Chem..

[112] Jahchan NS, Wang D, Bissell MJ, Luo K (2012). SnoN regulates mammary gland alveologenesis and onset of lactation by promoting prolactin/Stat5 signaling. Development.

[113] Wang Y (2017). BMP-7 enhances SnoN mRNA expression in renal tubular epithelial cells under high-glucose conditions. Mol. Med. Rep..

[114] Kodigepalli KM, Dutta PS, Bauckman KA, Nanjundan M (2013). SnoN/SkiL expression is modulated via arsenic trioxide-induced activation of the PI3K/AKT pathway in ovarian cancer cells. FEBS Lett..

[115] Park MA, Choi KC (2014). Effects of 4-nonylphenol and bisphenol A on stimulation of cell growth via disruption of the Transforming Growth Factor-β signaling pathway in ovarian cancer models. Chem. Res. Toxicol..

[116] Hamilton MP (2013). Identification of a pan-cancer oncogenic microRNA superfamily anchored by a central core seed motif. Nat. Commun..

[117] Xu H, Sun F, Li X, Sun L (2017). Down-regulation of miR-23a inhibits high glucose-induced EMT and renal fibrogenesis by up-regulation of SnoN. Hum. Cell.

[118] Dahl R, Wani B, Hayman MJ (1998). The Ski oncoprotein interacts with Skip, the human homolog of Drosophila Bx42. Oncogene.

[119] Prathapam T, Kuhne C, Hayman M, Banks L (2001). Ski interacts with the evolutionary conserved SNW domain of Skip. Nucleic Acids Res..

[120] Kim SS (2002). Structure of the retinal determination protein Dachshund reveals a DNA binding motif. Structure.

[121] Wilson JJ, Malakhova M, Zhang R, Joachimiak A, Hegde RS (2004). Crystal structure of the dachshund homology domain of human SKI. Structure.

[122] Wu JW (2002). Structural mechanism of SMAD4 recognition by the nuclear oncoprotein Ski: insights on Ski-mediated repression of TGF-β signaling. Cell.

[123] Stavnezer E, Brodeur D, Brennan LA (1989). The v-ski oncogene encodes a truncated set of c-ski coding exons with limited sequence and structural relatedness to v-myc. Mol. Cell Biol..

[124] Nagase T, Nomura N, Ishii S (1993). Complex formation between proteins encoded by the ski gene family. J. Biol. Chem..

[125] Nyman T (2010). The crystal structure of the dachshund domain of human SnoN reveals flexibility in the putative protein interaction surface. PLoS ONE.

[126] Walldén K, Nyman T, Hällberg BM (2017). SnoN stabilizes the SMAD3/SMAD4 protein complex. Sci. Rep..

[127] Heyman HC, Stavnezer E (1994). A carboxyl-terminal region of the ski oncoprotein mediates homodimerization as well as heterodimerization with the related protein SnoN. J. Biol. Chem..

[128] Zheng G (1997). High affinity dimerization by Ski involves parallel pairing of a novel bipartite α-helical domain. J. Biol. Chem..

[129] Cohen SB, Nicol R, Stavnezer E (1998). A domain necessary for the transforming activity of SnoN is required for specific DNA binding, transcriptional repression and interaction with TAF(II)110. Oncogene.

[130] Cohen SB, Zheng G, Heyman HC, Stavnezer E (1999). Heterodimers of the SnoN and Ski oncoproteins form preferentially over homodimers and are more potent transforming agents. Nucleic Acids Res..

[131] He J, Tegen SB, Krawitz AR, Martin GS, Luo K (2003). The transforming activity of Ski and SnoN is dependent on their ability to repress the activity of SMAD proteins. J. Biol. Chem..

[132] Pearson-White S (1993). SnoI, a novel alternatively spliced isoform of the ski protooncogene homolog, sno. Nucleic Acids Res..

[133] Pearson-White S, Crittenden R (1997). Proto-oncogene Sno expression, alternative isoforms and immediate early serum response. Nucleic Acids Res..

[134] Takaesu NT (2006). dSno facilitates baboon signaling in the Drosophila brain by switching the affinity of Medea away from Mad and toward dSMAD2. Genetics.

[135] Ramel MC (2007). Drosophila SnoN modulates growth and patterning by antagonizing TGF-β signalling. Mech. Dev..

[136] Band AM, Bjorklund M, Laiho M (2009). The phosphatidylinositol 3-kinase/Akt pathway regulates transforming growth factor-β signaling by destabilizing Ski and inducing SMAD7. J. Biol. Chem..

[137] Nagata M (2010). Identification of a phosphorylation site in c-Ski as serine 515. J. Biochem..

[138] Mosquera J (2011). Identification of Ski as a target for Aurora A kinase. Biochem. Biophys. Res. Commun..

[139] Rivas S (2016). The Ski protein is involved in the transformation pathway of aurora kinase A. J. Cell Biochem..

[140] Kajino T, Omori E, Ishii S, Matsumoto K, Ninomiya-Tsuji J (2007). TAK1 MAPK kinase kinase mediates Transforming Growth Factor-β signaling by targeting SnoN oncoprotein for degradation. J. Biol. Chem..

[141] Bonni S (2001). TGF-β induces assembly of a SMAD2-Smurf2 ubiquitin ligase complex that targets SnoN for degradation. Nat. Cell Biol..

[142] Stroschein SL, Bonni S, Wrana JL, Luo K (2001). SMAD3 recruits the anaphase-promoting complex for ubiquitination and degradation of SnoN. Genes Dev..

[143] Wan Y, Liu X, Kirschner MW (2001). The anaphase-promoting complex mediates TGF-beta signaling by targeting SnoN for destruction. Mol. Cell.

[144] Koinuma D (2003). Arkadia amplifies TGF-β superfamily signalling through degradation of SMAD7. EMBO J..

[145] Levy L (2007). Arkadia activates SMAD3/SMAD4-dependent transcription by triggering signal-induced SnoN degradation. Mol. Cell Biol..

[146] Nagano Y (2007). Arkadia induces degradation of SnoN and c-Ski to enhance transforming growth factor-β signaling. J. Biol. Chem..

[147] Stegmüller J, Huynh MA, Yuan Z, Konishi Y, Bonni A (2008). TGF-β-SMAD2 signaling regulates the Cdh1-APC/SnoN pathway of axonal morphogenesis. J. Neurosci..

[148] Koinuma D (2011). RB1CC1 protein positively regulates Transforming Growth Factor-β signaling through the modulation of Arkadia E3 ubiquitin ligase activity. J. Biol. Chem..

[149] Mizuide M (2003). Two short segments of SMAD3 are important for specific interaction of SMAD3 with c-Ski and SnoN. J. Biol. Chem..

[150] Fukasawa H (2006). Ubiquitin-dependent degradation of SnoN and Ski is increased in renal fibrosis induced by obstructive injury. Kidney Int..

[151] Zhou F (2017). USP4 inhibits SMAD4 monoubiquitination and promotes activin and BMP signaling. EMBO J..

[152] Hsu YH (2006). Sumoylated SnoN represses transcription in a promoter-specific manner. J. Biol. Chem..

[153] Wrighton KH (2007). Transforming growth factor-β-independent regulation of myogenesis by SnoN sumoylation. J. Biol. Chem..

[154] Netherton SJ, Bonni S (2010). Suppression of TGFβ-induced epithelial-mesenchymal transition like phenotype by a PIAS1 regulated sumoylation pathway in NMuMG epithelial cells. PLoS ONE.

[155] Ikeuchi Y (2014). TIF1γ protein regulates epithelial-mesenchymal transition by operating as a small ubiquitin-like modifier (SUMO) E3 ligase for the transcriptional regulator SnoN1. J. Biol. Chem..

[156] Ding B, Sun Y, Huang J (2012). Overexpression of SKI oncoprotein leads to p53 degradation through regulation of MDM2 protein sumoylation. J. Biol. Chem..

[157] Krakowski AR, Laboureau J, Mauviel A, Bissell MJ, Luo K (2005). Cytoplasmic SnoN in normal tissues and nonmalignant cells antagonizes TGF-β signaling by sequestration of the SMAD proteins. Proc. Natl Acad. Sci. USA.

[158] Nagata M (2006). Nuclear and cytoplasmic c-Ski differently modulate cellular functions. Genes Cell.

[159] Kokura K (2003). The Ski-binding protein C184M negatively regulates tumor growth factor-β signaling by sequestering the SMAD proteins in the cytoplasm. J. Biol. Chem..

[160] Rajagopal R, Ishii S, Beebe DC (2007). Intracellular mediators of transforming growth factor beta superfamily signaling localize to endosomes in chicken embryo and mouse lenses in vivo. BMC Cell Biol..

[161] Ferrand N, Atfi A, Prunier C (2010). The oncoprotein c-ski functions as a direct antagonist of the Transforming Growth Factor-β type I receptor. Cancer Res..

[162] Javelaud D (2011). Efficient TGF-β/Smad signaling in human melanoma cells associated with high c-Ski/SnoN expression. Mol. Cancer.

[163] Reed JA (2001). Cytoplasmic localization of the oncogenic protein Ski in human cutaneous melanomas in vivo: functional implications for transforming growth factor β signaling. Cancer Res..

[164] Jahchan NS, You YH, Muller WJ, Luo K (2010). Transforming Growth Factor-β regulator SnoN modulates mammary gland branching morphogenesis, postlactational involution, and mammary tumorigenesis. Cancer Res..

[165] Jahchan NS, Ouyang G, Luo K (2013). Expression profiles of SnoN in normal and cancerous human tissues support its tumor suppressor role in human cancer. PLoS ONE.

[166] Ji X (2017). Vitamin C deficiency exacerbates diabetic glomerualr injury through activation of transforming growth factor-β signaling. Biochim. Biophys. Acta.

[167] Atanasoski S (2004). The protooncogene Ski controls Schwann cell proliferation and myelination. Neuron.

[168] Jacob C, Grabner H, Atanasoski S, Suter U (2008). Expression and localization of Ski determine cell type-specific TGFβ signaling effects on the cell cycle. J. Cell. Biol..

[169] Cunnington RH (2011). Antifibrotic properties of c-Ski and its regulation of cardiac myofibroblast phenotype and contractility. Am. J. Physiol. Cell Physiol..

[170] Zhu Q (2016). SnoN Antagonizes the hippo kinase complex to promote TAZ signaling during breast carcinogenesis. Dev. Cell.

[171] Vázquez-Victorio G (2015). Novel regulation of Ski protein stability and endosomal sorting by actin cytoskeleton dynamics in hepatocytes. J. Biol. Chem..

[172] Caligaris C (2015). Actin-cytoskeleton polymerization differentially controls the stability of Ski and SnoN co-repressors in normal but not in transformed hepatocytes. Biochim. Biophys. Acta.

[173] Vázquez-Victorio G, González-Espinosa C, Espinosa-Riquer ZP, Macías-Silva M (2016). GPCRs and actin-cytoskeletal dynamics. Method Cell Biol..

[174] Macdonald M (2004). Control of cell cycle-dependent degradation of c-Ski proto-oncoprotein by Cdc34. Oncogene.

[175] Marcelain K, Hayman MJ (2005). The Ski oncoprotein is upregulated and localized at the centrosomes and mitotic spindle during mitosis. Oncogene.

[176] Marcelain K (2012). Chromosomal instability in mouse embryonic fibroblasts null for the transcriptional co-repressor Ski. J. Cell Physiol..

[177] Zieba A (2012). Intercellular variation in signaling through the TGF-β pathway and its relation to cell density and cell cycle phase. Mol. Cell Prot..

[178] Shinagawa T, Dong HD, Xu M, Maekawa T, Ishii S (2000). The sno gene, which encodes a component of the histone deacetylase complex, acts as a tumor suppressor in mice. EMBO J..

[179] Pearson-White S, McDuffie M (2003). Defective T-cell activation is associated with augmented transforming growth factor β sensitivity in mice with mutations in the Sno gene. Mol. Cell Biol..

[180] Zhu Q, Kim YH, Wang D, Oh SP, Luo K (2013). SnoN facilitates ALK1-SMAD1/5 signaling during embryonic angiogenesis. J. Cell Biol..

[181] Shinagawa T, Ishii S (2003). Generation of Ski-knockdown mice by expressing a long double-strand RNA from an RNA polymerase II promoter. Genes Dev..

[182] McGannon P, Miyazaki Y, Gupta PC, Traboulsi EI, Colmenares C (2006). Ocular abnormalities in mice lacking the Ski proto-oncogene. Invest. Ophthalmol. Vis. Sci..

[183] Lyons GE (1994). Protooncogene c-ski is expressed in both proliferating and postmitotic neuronal populations. Dev. Dyn..

[184] Pot I, Ikeuchi Y, Bonni A, Bonni S (2010). SnoN: Bridging neurobiology and cancer biology. Curr. Mol. Med..

[185] Bonni S, Bonni A (2012). SnoN signaling in proliferating cells and postmitotic neurons. FEBS Lett..

[186] Amaravadi LS, Neff AW, Sleeman JP, Smith RC (1997). Autonomous neural axis formation by ectopic expression of the protooncogene c-ski. Dev. Biol..

[187] Zhou K (2017). Spatiotemporal expression of Ski after rat spinal cord injury. Neuroreport.

[188] Chang C, Harland RM (2007). Neural induction requires continued suppression of both SMAD1 and SMAD2 signals during gastrulation. Development.

[189] Berk M, Desai SY, Heyman HC, Colmenares C (1997). Mice lacking the ski proto-oncogene have defects in neurulation, craniofacial, patterning, and skeletal muscle development. Genes Dev..

[190] Colmenares C (2002). Loss of the SKI proto-oncogene in individuals affected with 1p36 deletion syndrome is predicted by strain-dependent defects in Ski^-/-^ mice. Nat. Genet..

[191] Huynh MA (2011). An isoform-specific SnoN1-FOXO1 repressor complex controls neuronal morphogenesis and positioning in the mammalian brain. Neuron.

[192] Do JL, Bonni A, Tuszynski MH (2013). SnoN facilitates axonal regeneration after spinal cord injury. PLoS ONE.

[193] Pearson-White S (1995). The ski/sno protooncogene family in hematopoietic development. Blood.

[194] Singbrant S (2014). The SKI proto-oncogene enhances the in vivo repopulation of hematopoietic stem cells and causes myeloproliferative disease. Haematologica.

[195] Dahl R, Kieslinger M, Beug H, Hayman MJ (1998). Transformation of hematopoietic cells by the Ski oncoprotein involves repression of retinoic acid receptor signaling. Proc. Natl Acad. Sci. USA.

[196] Namciu S, Lieberman MA, Stavnezer E (1994). Induction of the c-ski proto-oncogene by phorbol ester correlates with induction of megakaryocyte differentiation. Oncogene.

[197] Li C (2014). PMA induces SnoN proteolysis and CD61 expression through an autocrine mechanism. Cell Signal..

[198] Engert JC, Servaes S, Sutrave P, Hughes SH, Rosenthal N (1995). Activation of a muscle-specific enhancer by the Ski proto-oncogene. Nucleic Acids Res..

[199] Ambrose MR, Bottazzi ME, Goodenow MM (1995). Expression of the c-ski proto-oncogene during cell cycle arrest and myogenic differentiation. DNA Cell Biol..

[200] Namciu S (1995). Enhanced expression of mouse c-ski accompanies terminal skeletal muscle differentiation in vivo and in vitro. Dev. Dyn..

[201] Colmenares C, Stavnezer E (1989). The ski oncogene induces muscle differentiation in quail embryo cells. Cell.

[202] Colmenares C, Teumer JK, Stavnezer E (1991). Transformation-defective v-ski induces MyoD and myogenin expression but not myotube formation. Mol. Cell Biol..

[203] Ichikawa K, Nagase T, Ishii S, Asano A, Mimura N (1997). Trans-regulation of myogenin promoter/enhancer activity by c-ski during skeletal-muscle differentiation: the C-terminus of the c-Ski protein is essential for transcriptional regulatory activity in myotubes. Biochem. J..

[204] Sutrave P, Kelly AM, Hughes SH (1990). Ski can cause selective growth of skeletal muscle in transgenic mice. Genes Dev..

[205] Costelli P (2003). Reduced protein degradation rates and low expression of proteolytic systems support skeletal muscle hypertrophy in transgenic mice overexpressing the c-ski oncogene. Cancer Lett..

[206] Lana DP, Leferovich JM, Kelly AM, Hughes SH (1996). Selective expression of a ski transgene affects IIb fast muscles and skeletal structure. Dev. Dyn..

[207] Bowen RA (1994). Transgenic cattle resulting from biopsied embryos: expression of c-ski in a transgenic calf. Biol. Reprod..

[208] Soeta C (2001). Possible role for the c-ski gene in the proliferation of myogenic cells in regenerating skeletal muscles of rats. Dev. Growth Differ..

[209] Kano K (1998). C. Skeletal muscles of transgenic mice expressing human SnoN, a homologue of c-ski. J. Reprod. Dev..

[210] Wang Y (2016). Smad2 and Smad3 regulate chondrocyte proliferation and differentiation in the growth plate. PLoS Genet..

[211] Leong GM (2010). The Ski proto-oncogene regulates body composition and suppresses lipogenesis. Int. J. Obes..

[212] Ye F (2011). Peroxisome proliferator-activated receptor gamma (PPARγ) mediates a Ski oncogene-induced shift from glycolysis to oxidative energy metabolism. J. Biol. Chem..

[213] Diaz M (2012). Ski overexpression in skeletal muscle modulates genetic programs that control susceptibility to diet-induced obesity and insulin signaling. Obesity.

[214] Huang W (2014). The proteasome inhibitor, MG132, attenuates diabetic nephropathy by inhibiting SnoN degradation in vivo and in vitro. Biomed. Res. Int..

[215] Liu L (2016). Oxymatrine inhibits renal tubular EMT induced by high glucose via upregulation of SnoN and inhibition of TGF-β1/SMAD signaling pathway. PLoS ONE.

[216] Sengupta S, Jana S, Biswas S, Mandal PK, Bhattacharyya A (2013). Cooperative involvement of NFAT and SnoN mediates transforming growth factor-β (TGF-β) induced EMT in metastatic breast cancer (MDA-MB 231) cells. Clin. Exp. Metastas..

[217] Esposito C (2009). The antifibrogenic effect of hepatocyte growth factor (HGF) on renal tubular (HK-2) cells is dependent on cell growth. Growth Factors.

[218] Li X (2016). The downregulation of SnoN expression in human renal proximal tubule epithelial cells under high-glucose conditions is mediated by an increase in Smurf2 expression through TGF-β1 signaling. Int. J. Mol. Med..

[219] Tang H (2016). MAD2B-mediated SnoN downregulation is implicated in fibroblast activation and tubulointerstitial fibrosis. Am. J. Physiol. Ren. Physiol..

[220] Vázquez-Macías A, Ruiz-Mendoza AB, Fonseca-Sánchez MA, Briones-Orta MA, Macías-Silva M (2005). Downregulation of Ski and SnoN co-repressors by anisomycin. FEBS Lett..

[221] Macías-Silva M, Vázquez-Victorio G, Hernández-Damián J (2010). Anisomycin is a multifunctional drug: more than just a tool to inhibit protein synthesis. Curr. Chem. Biol..

[222] Hernández-Damián J (2013). Downregulation of SnoN oncoprotein induced by antibiotics anisomycin and puromycin positively regulates transforming growth factor-β signals. Biochim. Biophys. Acta.

[223] Ehnert S (2012). Transforming growth factor β1 inhibits bone morphogenic protein (BMP)-2 and BMP-7 signaling via upregulation of Ski-related novel protein N (SnoN): possible mechanism for the failure of BMP therapy?. BMC Med..

[224] Pan D, Zhu Q, Conboy MJ, Conboy IM, Luo K (2012). SnoN activates p53 directly to regulate aging and tumorigenesis. Aging Cell.

[225] Makino Y (2017). Repression of Smad3 by Stat3 and c-Ski/SnoN induces gefitinib resistance in lung adenocarcinoma. Biochem. Biophys. Res. Commun..

[226] López-Novoa JM, Nieto MA (2009). Inflammation and EMT: an alliance towards organ fibrosis and cancer progression. EMBO Mol. Med..

[227] Tan EJ, Olsson AK, Moustakas A (2015). Reprogramming during epithelial to mesenchymal transition under the control of TGFβ. Cell Adh. Migr..

[228] Moustakas A, Heldin CH (2016). Mechanisms of TGFβ-induced epithelial-mesenchymal transition. J. Clin. Med..

[229] Macías-Silva M, Li W, Leu JI, Crissey MA, Taub R (2002). Up-regulated transcriptional repressors SnoN and Ski bind SMAD proteins to antagonize transforming growth factor-β signals during liver regeneration. J. Biol. Chem..

[230] Zhang Q, Zhou J, Ge H, Cheng B (2013). Tgif1 and SnoN modified chondrocytes or stem cells for tendon-bone insertion regeneration. Med. Hypotheses.

[231] Liu X (2006). Expression and possible mechanism of c-ski, a novel tissue repair-related gene during normal and radiation-impaired wound healing. Wound Repair Regen..

[232] Peng Y (2016). Comparative evaluation of the wound-healing potency of recombinant bFGF and ski gene therapy in rats. Growth Factors.

[233] Li J (2013). c-Ski inhibits the proliferation of vascular smooth muscle cells via suppressing SMAD3 signaling but stimulating p38 pathway. Cell Signal..

[234] Yang J, Zhang X, Li Y, Liu Y (2003). Downregulation of SMAD transcriptional corepressors SnoN and Ski in the fibrotic kidney: an amplification mechanism for TGF-β1 signaling. J. Am. Soc. Nephrol..

[235] Liu R (2012). SnoN as a key regulator of the high glucose-induced epithelial-mesenchymal transition in cells of the proximal tubule. Kidney Blood Press Res..

[236] Tan R (2006). Downregulation of SnoN expression in obstructive nephropathy is mediated by an enhanced ubiquitin-dependent degradation. J. Am. Soc. Nephrol..

[237] Xu Z, Diao Z, Liu R, Liu W (2017). Molecular mechanism of smurf2 in regulating the expression of SnoN in diabetic nephropathy. Mol. Med. Rep..

[238] Liu L (2017). SnoN upregulation ameliorates renal fibrosis in diabetic nephropathy. PLoS ONE.

[239] Chen J (2013). Docosahexaenoic acid (DHA) ameliorates paraquat-induced pulmonary fibrosis in rats possibly through up-regulation of SMAD 7 and SnoN. Food Chem. Toxicol..

[240] Wang J (2017). The role of c-SKI in regulation of TGF-β-induced human cardiac fibroblast proliferation and ECM protein expression. J. Cell Biochem..

[241] Zeglinski MR (2016). Chronic expression of Ski induces apoptosis and represses autophagy in cardiac myofibroblasts. Biochim. Biophys. Acta.

[242] Kishore R (2013). Bone marrow progenitor cell therapy-mediated paracrine regulation of cardiac miRNA-155 modulates fibrotic response in diabetic hearts. PLoS ONE.

[243] Cunnington RH (2014). The Ski-Zeb2-Meox2 pathway provides a novel mechanism for regulation of the cardiac myofibroblast phenotype. J. Cell Sci..

[244] Reyes-Gordillo K (2014). Mechanisms of action of acetaldehyde in the up-regulation of the human alpha2(I) collagen gene in hepatic stellate cells: key roles of Ski, SMAD3, SMAD4, and SMAD7. Am. J. Pathol..

[245] Jinnin M, Ihn H, Mimura Y, Asano Y, Tamaki K (2007). Involvement of the constitutive complex formation of c-Ski/SnoN with SMADs in the impaired negative feedback regulation of transforming growth factor β signaling in scleroderma fibroblasts. Arthr. Rheum..

[246] Li P (2011). Ski, a modulator of wound healing and scar formation in the rat skin and rabbit ear. J. Pathol..

[247] Obenauf AC, Massagué J (2015). Surviving at a distance: organ specific metastasis. Trends Cancer.

[248] David CJ (2016). TGF-β Tumor Suppression through a Lethal EMT. Cell.

[249] Nieto MA, Huang RY, Jackson RA, Thiery JP (2016). Emt: 2016. Cell.

[250] Lebrun JJ (2012). The dual role of TGFβ in human cancer: from tumor suppression to cancer metastasis. ISRN Mol. Biol..

[251] Colmenares C, Sutrave P, Hughes SH, Stavnezer E (1991). Activation of the c-ski oncogene by overexpression. J. Virol..

[252] Zheng G, Teumer J, Colmenares C, Richmond C, Stavnezer E (1997). Identification of a core functional and structural domain of the v-Ski oncoprotein responsible for both transformation and myogenesis. Oncogene.

[253] Fumagalli S, Doneda L, Nomura N, Larizza L (1993). Expression of the c-ski proto-oncogene in human melanoma cell lines. Melanoma Res..

[254] Imoto I (2001). SNO is a probable target for gene amplification at 3q26 in squamous-cell carcinomas of the esophagus. Biochem. Biophys. Res. Commun..

[255] Zhang F (2003). Ski-related novel protein N (SnoN), a negative controller of transforming growth factor-β signaling, is a prognostic marker in estrogen receptor-positive breast carcinomas. Cancer Res..

[256] Buess M (2004). Amplification of SKI is a prognostic marker in early colorectal cancer. Neoplasia.

[257] Fukuchi M (2004). Increased expression of c-Ski as a co-repressor in transforming growth factor-β signaling correlates with progression of esophageal squamous cell carcinoma. Int. J. Cancer.

[258] Kronenwett R (2005). Distinct molecular phenotype of malignant CD34(+) hematopoietic stem and progenitor cells in chronic myelogenous leukemia. Oncogene.

[259] Poser I, Rothhammer T, Dooley S, Weiskirchen R, Bosserhoff AK (2005). Characterization of Sno expression in malignant melanoma. Int. J. Oncol..

[260] Edmiston JS, Yeudall WA, Chung TD, Lebman DA (2005). Inability of transforming growth factor-β to cause SnoN degradation leads to resistance to Transforming Growth Factor-β-induced growth arrest in esophageal cancer cells. Cancer Res..

[261] Heider TR, Lyman S, Schoonhoven R, Behrns KE (2007). Ski promotes tumor growth through abrogation of Transforming Growth Factor-β signaling in pancreatic cancer. Ann. Surg..

[262] Bravou V (2009). TGF-β repressors SnoN and Ski are implicated in human colorectal carcinogenesis. Cell. Oncol..

[263] Boone B, Haspeslagh M, Brochez L (2009). Clinical significance of the expression of c-Ski and SnoN, possible mediators in TGF-β resistance in primary cutaneous melanoma. J. Dermatol. Sci..

[264] Chen D (2009). SKI knockdown inhibits human melanoma tumor growth in vivo. Pigment. Cell. Melanoma Res..

[265] Kiyono K (2009). c-Ski overexpression promotes tumor growth and angiogenesis through inhibition of transforming growth factor-β signaling in diffuse-type gastric carcinoma. Cancer Sci..

[266] O TM (2009). Differential expression of SKI oncogene protein in hemangiomas. Otolaryngol. Head Neck Surg..

[267] Takahata M (2009). SKI and MEL1 cooperate to inhibit transforming growth factor-beta signal in gastric cancer cells. J. Biol. Chem..

[268] Wang P (2009). Dual role of Ski in pancreatic cancer cells: tumor-promoting versus metastasis-suppressive function. Carcinogenesis.

[269] Nakao T (2011). Expression of thrombospondin-1 and Ski are prognostic factors in advanced gastric cancer. Int. J. Clin. Oncol..

[270] Bravou V (2012). Transforming growth factor β repressor, SnoN, is overexpressed in human gastrointestinal stromal tumors. J. Buon..

[271] Chen Y, Pirisi L, Creek KE (2013). Ski protein levels increase during in vitro progression of HPV16-immortalized human keratinocytes and in cervical cancer. Virology.

[272] Liu C (2015). The influence of SnoN gene silencing by siRNA on the cell proliferation and apoptosis of human pancreatic cancer cells. Diagn. Pathol..

[273] Shinagawa T (2001). Increased susceptibility to tumorigenesis of ski-deficient heterozygous mice. Oncogene.

[274] Villanacci V (2008). Ski/SnoN expression in the sequence metaplasia-dysplasia-adenocarcinoma of Barrett’s esophagus. Hum. Pathol..

[275] Chia JA (2006). SnoN expression is differently regulated in microsatellite unstable compared with microsatellite stable colorectal cancers. BMC Cancer.

[276] Hagerstrand D (2013). Systematic interrogation of 3q26 identifies TLOC1 and SKIL as cancer drivers. Cancer Discov..

[277] Sheu JJC (2009). Chromosome 3p12.3-p14.2 and 3q26.2-q26.32 are genomic markers for prognosis of advanced nasopharyngeal carcinoma. Cancer Epidemiol. Biomark. Prev..

[278] Nanjundan M (2007). Amplification of MDS1/EVI1 and EVI1, located in the 3q26.2 amplicon, is associated with favorable patient prognosis in ovarian cancer. Cancer Res..

[279] Nanjundan M (2008). Overexpression of SnoN/SkiL, amplified at the 3q26.2 locus, in ovarian cancers: a role in ovarian pathogenesis. Mol. Oncol..

[280] Yang H (2015). Ski prevents TGF-β-induced EMT and cell invasion by repressing SMAD-dependent signaling in non-small cell lung cancer. Oncol. Rep..

[281] Song L (2016). Ski modulate the characteristics of pancreatic cancer stem cells via regulating sonic hedgehog signaling pathway. Tumor Biol..

[282] Ritter M (2006). A. Inhibition of retinoic acid receptor signaling by Ski in acute myeloid leukemia. Leukemia.

[283] Wang L (2013). c-Ski activates cancer-associated fibroblasts to regulate breast cancer cell invasion. Mol. Oncol..

[284] Schweighofer CD (2011). A two-gene signature, SKI and SLAMF1, predicts time-to-treatment in previously untreated patients with chronic lymphocytic leukemia. PLoS ONE.

[285] Theohari I (2012). Differential effect of the expression of TGF-β pathway inhibitors, SMAD-7 and Ski, on invasive breast carcinomas: relation to biologic behavior. APMIS.

[286] Gallo-Oller G (2016). P144, a transforming growth factor beta inhibitor peptide, generates antitumoral effects and modifies SMAD7 and SKI levels in human glioblastoma cell lines. Cancer Lett..

[287] Zhu Q (2007). Dual role of SnoN in mammalian tumorigenesis. Mol. Cell Biol..

[288] Chanda A (2017). Identification of the SUMO E3 ligase PIAS1 as a potential survival biomarker in breast cancer. PLoS ONE.

[289] Zhang X, Egawa K, Xie Y, Ihn H (2009). The expression of SnoN in normal human skin and cutaneous keratinous neoplasms. Int. J. Dermatol..

[290] Zhu X (2013). 576 kb deletion in 1p36.33-p36.32 containing SKI is associated with limb malformation, congenital heart disease and epilepsy. Gene.

[291] Schepers D (2015). The SMAD-binding domain of SKI: a hotspot for de novo mutations causing Shprintzen-Goldberg syndrome. Eur. J. Hum. Genet..

[292] Carmignac V (2012). In-frame nutations in exon 1 of SKI cause dominant Shpritzen-Goldberg syndrome. Am. J. Hum. Genet..

[293] Doyle AJ (2012). Mutations in the TGF-β repressor SKI cause Shprintzen-Goldberg syndrome with aortic aneurysm. Nat. Genet..

[294] Colak S, ten Dijke P (2016). Targeting TGF-β signaling in cancer. Trends Cancer.

[295] Inoue Y, Imamura T (2008). Regulation of TGF-β family signaling by E3 ubiquitin ligases. Cancer Sci..

[296] Wang W, Liu C, Wang Y, Cao L (2013). Effects of the downregulation of SnoN expression on HepG2 cell proliferation and apoptosis. Mol. Med. Rep..

[297] Vázquez-Victorio, G., Rosales-Alvarez, R. E., Ríos-López, D. G., Tecalco-Cruz, A. C., Macías-Silva, M. In *Advances in health and disease*, Vol. 1 (ed Duncan, L. T.) (Nova Science Publishers: New York, USA, NY, 2017) pp 63–135.

